# Synthesis of coumarin derivatives and investigation of their inhibitory effects on lung cancer cell motility

**DOI:** 10.1038/s41598-022-26212-z

**Published:** 2022-12-14

**Authors:** Rui Zhou, Young Hyun Yu, Hangun Kim, Hyung-Ho Ha

**Affiliations:** College of Pharmacy, Sunchon National University, 255 Jungang-Ro, Sunchon, Jeonnam 57922 Republic of Korea

**Keywords:** Cancer, Chemical biology

## Abstract

Based on the pharmaceutical potentials of coumarins, which have antitumor activity, we synthesized new coumarin derivatives and evaluated their biological activities. The new coumarin derivatives were chemically synthesized from 4-hydroxycoumarin, and their structures were confirmed by nuclear magnetic resonance data. Ten of the synthesized compounds were investigated for antimetastatic activity against lung carcinoma cells. Several of the tested compounds showed good to mild inhibitory effects on lung cancer cell motility. There were no cytotoxic effects related to the use of these compounds. 4-Hydroxycoumarin derivatives, **4h** and **4i**, elicited the significant inhibitory effect on lung cancer cell motility by suppressing expression of the epithelial–mesenchymal transition markers N-cadherin, Snail, and Twist.

## Introduction

Lung adenocarcinoma is the most malignant diagnosed cancer and is the leading cause of cancer death in men worldwide, followed by colorectal and liver cancer^1,2^. Lung cancer has various preferential sites for metastasis, such as the liver, brain, and bones^3^. Patients with metastatic lung adenocarcinoma have a poor prognosis and usually survive for fewer than 5 years^4^. The epithelial–mesenchymal transition (EMT) is a common mechanism underlying lung cancer cell motility and progression^5^. It facilitates metastasis by promoting lung cancer cell invasion, migration, and resistance to apoptotic stimuli. Therefore, inhibition of the EMT suppresses metastasis and progression of lung cancer^6^.

Coumarins, which are well-known condensed benzoheterocycles, are used as medicinal chemicals because they are easily synthetically transformed into a large variety of functionalized structures^7^. They have various purposes, such as synthesis of medicines^8^; laser dyes; and compounds with antifungal^9^, antibacterial^10^, anti-inflammatory^11^, and anticancer activities^11,12^. Recently, coumarin derivatives were reported to have inhibitory effects on prostate cancer, colon cancer, gastric cancer, and pancreatic cancer, mainly by regulating cell proliferation and apoptosis. Coumarin derivative esculetin inhibits the proliferation of human prostate cancer cells by inducing apoptosis and arresting the cell cycle^13^. 5-Geranyloxy-7-methoxycoumarin inhibits colon cancer cell growth via induction of apoptosis^14^. Coumarin-indole derivative MY-413 induces gastric cancer cell apoptosis and inhibits proliferation through MAPK signaling pathway^15^. Coumarin derivatives (12a–x) functions as NEDD8 activating enzyme inhibitors and induces apoptosis in pancreatic cancer cells^16^. Due to their many uses, it has become important to synthesize coumarins or their derivatives with conventional drugs by various reactions^17,18^. Various synthetic methods for coumarins and their derivatives have been used to insert various substituents into specific areas of the coumarin structure, thereby allowing mechanistic exploration of their anticancer activities. Herein, we summarize the inhibitory effects of coumarin derivatives on lung cancer cell motility.

## Results and discussion

### Chemistry

**Figure 1:**
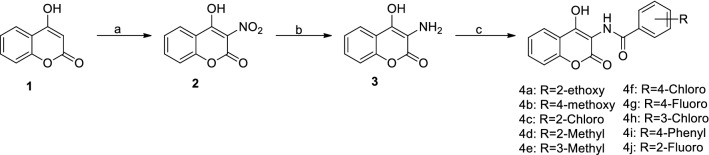
Synthesis pathway of coumarin analogues. Reagents and conditions: (**a**) HNO_3_, NaNO_2_, AcOH, 1 h, 60 °C; (**b**) H_2_, Pd/C, EtOH, 8 h, rt; (**c**) Benzoyl chloride, Pyridine, CH_2_Cl_2_, 8 h, rt.

4-Hydroxycoumarin is an effective drug for small cell lung cancer^15^. Therefore, we used 4-hydroxycoumarin as a material to chemically synthesize 3-nitro-4-hydroxycoumarin (**2**) in the presence of HNO_3_ at 60 °C in an oil bath and then 3-amino-4-hydroxycoumarin (**3**) in the presence of Pd/C, and in a H_2_ atmosphere for 8 h (Fig. 1).

**Figure 2:**
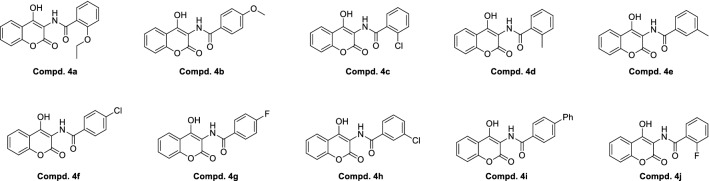
Structure of Aminocoumarin derivatives (**4a**–**4j**).

3-Amino-4-hydroxycoumarin (**3**) has an amino group (NH_2_), which is a stable functional site. Variously substituted acid chlorides (**4a**–**4j**) were selected as the linker via a carboxyl group (–COOH). Ten 3-amidocoumarins (**4a**–**4j**) were synthesized by a reaction involving formation of a peptide bond (–CO–NH–). A peptide bond was used due to its resonance stabilization and readiness to undergo chemical reactions. The structures of different aminocoumarin derivatives are presented in Fig. 2.

### Biological evaluation

**Figure 3:**
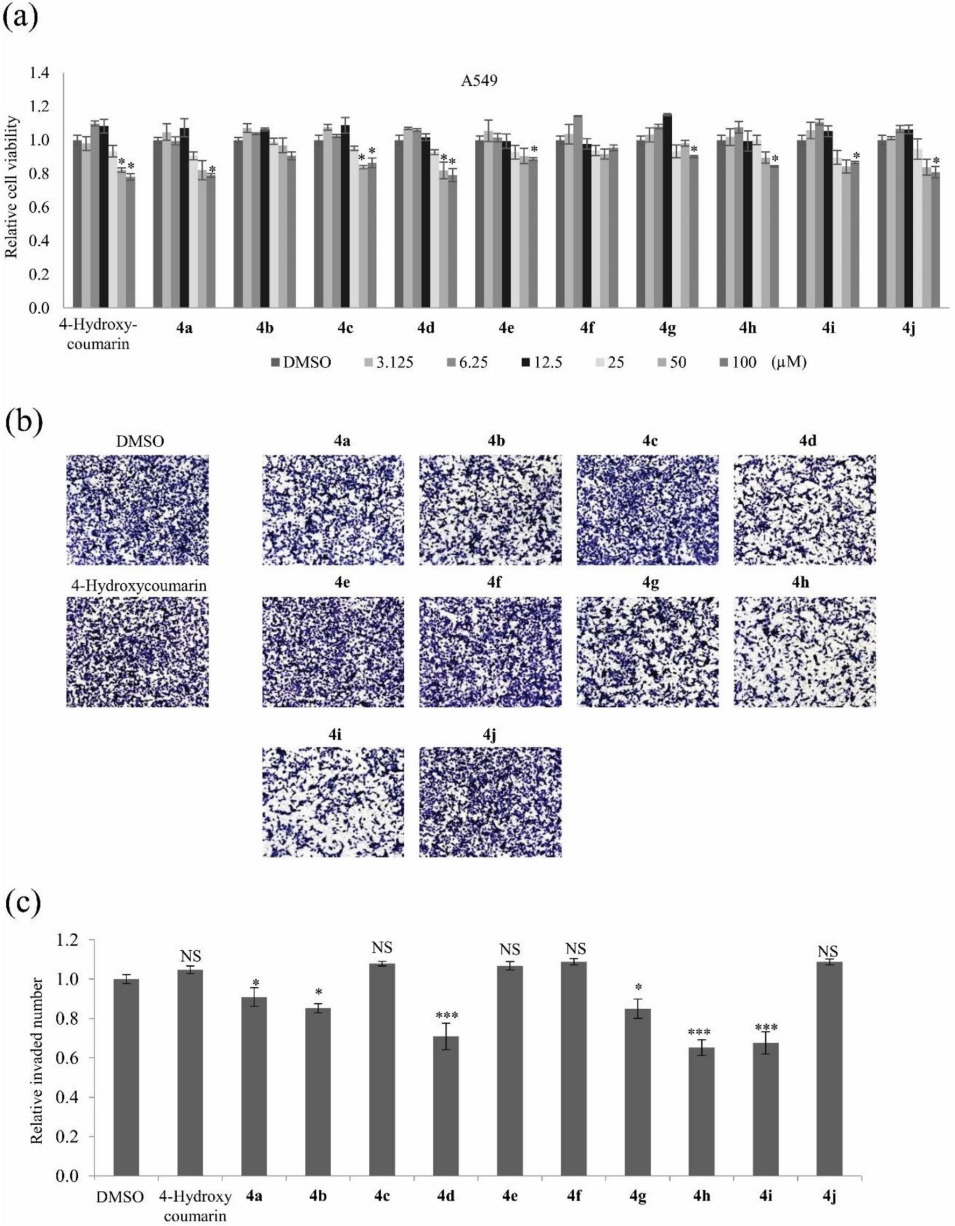
The effects of 4-hydroxycoumarin and its derivatives **4a**–**4j** on invasion of A549 cells. (**a**) Relative viabilities of A549 cells after treatment with 4-hydroxycoumarin and its derivatives **4a**–**4j** for 48 h at concentrations ranging from 3.125 to 100 μM measured by the MTT assay. (**b**) Representative images of invasion assays of A549 cells treated with 4-hydroxycoumarin and its derivatives **4a**–**4j** at a concentration of 5 μM. (**c**) Quantitative analysis of invaded cell numbers after each treatment. Representative images from three independent experiments are shown (n = 3). Data represent the mean ± SD. *p < 0.05; ***p < 0.001; NS, no significant difference compared with dimethyl sulfoxide (DMSO)-treated A549 cells.

Cancer metastasis accounts for much of the morbidity and is associated with ~ 90% of the cancer-associated deaths^19^. According to the GLOBOCAN analysis, lung cancer is one of the most malignant types with metastasis, with a higher mortality rate of around 18.4%^20^. The motility of cancer cells contributes to tumor metastasis with several stages, including invading across the basement membranes, migration to blood, intravasation and movement into distant organs^21^. Cell migration and invasion are relevant phenotypes when studying the effect of novel therapeutic drugs against metastasis in cancer development and progression^22^. This study aimed to elucidate whether chemically synthesized coumarin derivatives affect lung cancer cell motility. To determine the cytotoxic effects of all the compounds on lung cancer cells, viability of A549, H460, and H1975 cells were evaluated via the MTT assays. Treatment with 3.125–20 μM of 4-hydroxycoumarin and its derivatives **4a**–**4j** did not affect the viabilities of A549, H460, and H1975 cells. Treatment with 50–100 μM of 4-hydroxycoumarin and its derivatives **4a**–**4j** inhibited the cell viabilities by ~ 25% in A549, H460, and H1975 cells (Fig. 3a and Supplementary Fig. S1). An invasion assay was performed to examine their effects on A549 cell motility. Cells were treated with 4-hydroxycoumarin and its derivatives **4a**–**4j** at nontoxic concentration of 5 μM for 24 h. Treatment with compounds **4d**, **4h**, and **4i** significantly inhibited A549 cell invasion by ~ 40%, ~ 50%, and ~ 40% (*p* < 0.0001), respectively (Fig. 3b,c).

**Figure 4:**
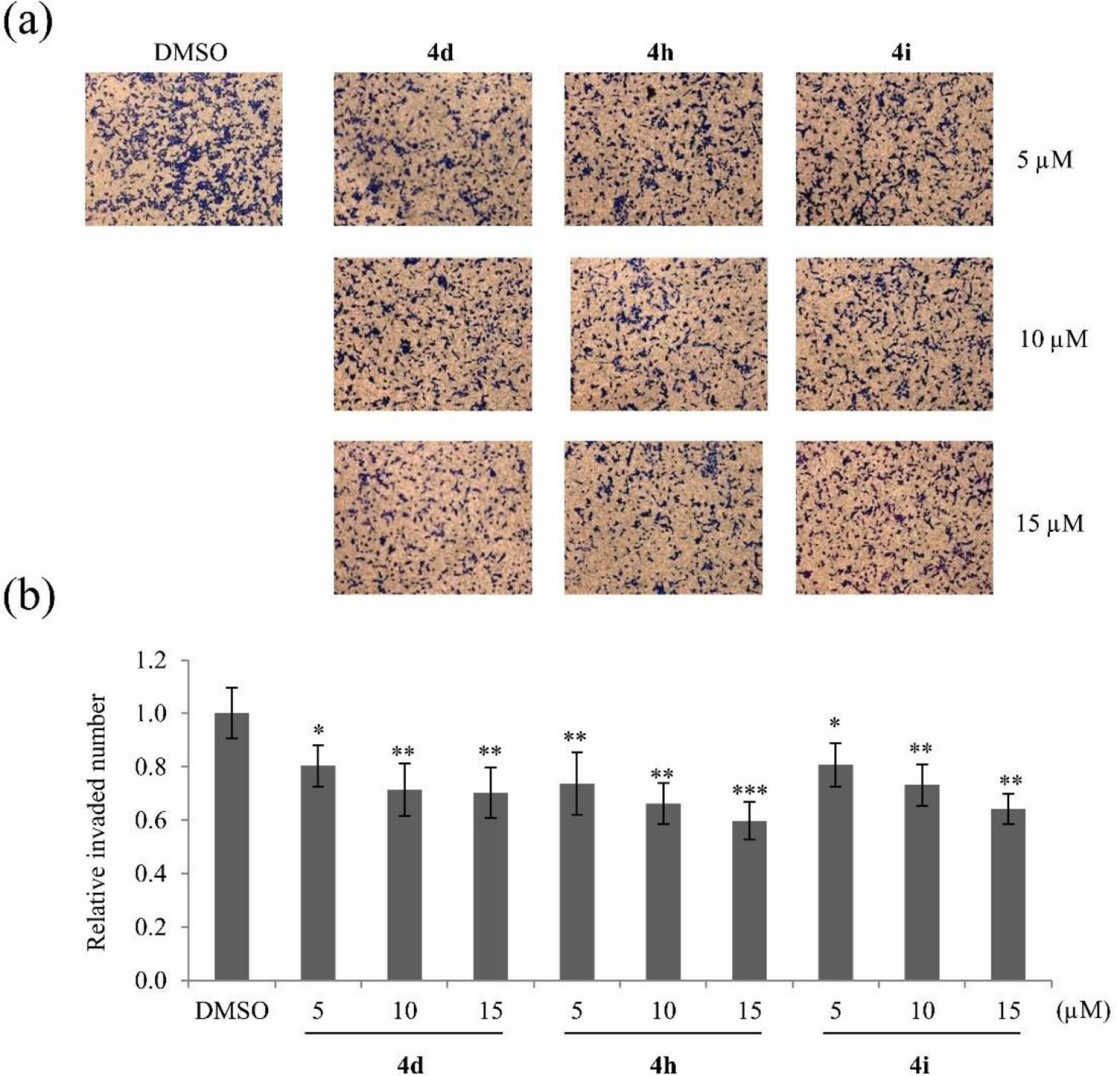
Suppression of A549 cell invasion by compounds **4d**, **4h**, and **4i**. (**a**) Representative images of invaded A549 cells after treatment with various nontoxic concentrations (5, 10, and 15 μM) of compounds **4d**, **4h**, and **4i** for 24 h. (**b**) Quantitative analysis of invaded cell numbers in each group. Representative images from three independent experiments are shown (n = 3). Data represent the mean ± SD. *p < 0.05; **p < 0.01; ***p < 0.001 compared with DMSO-treated A549 cells.

To elucidate how these three compounds inhibit cancer cell motility at noncytotoxic concentrations, cells were mainly treated with nontoxic concentrations at 5, 10, or 15 μM of compounds **4d**, **4h**, and **4i** in subsequent experiments. Treatment with compounds **4d**, **4h**, and **4i** dose-dependently decreased the number of invaded A549 cells (Fig. 4).

**Figure 5:**
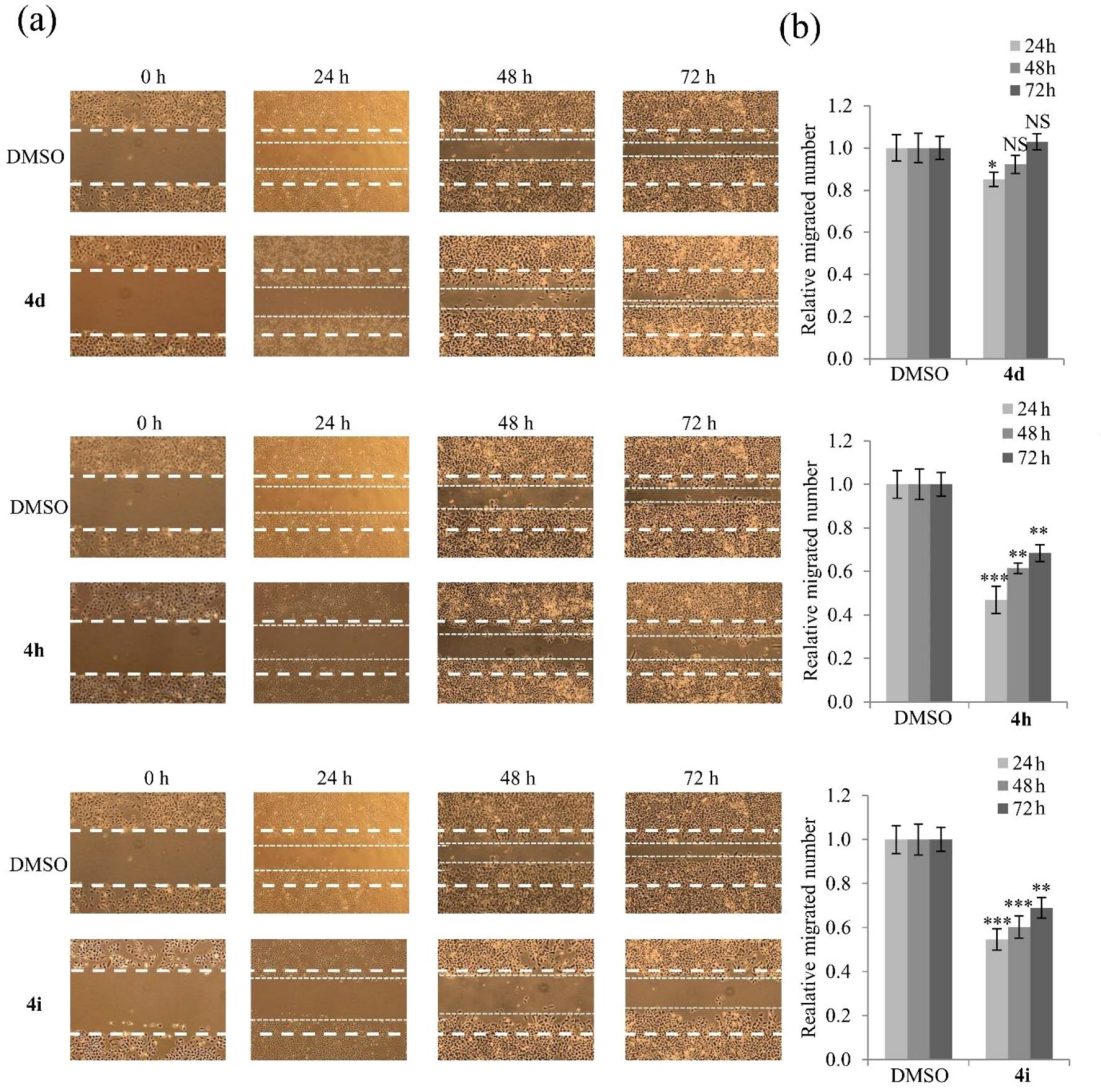
Inhibition of A549 cell migration by compounds **4d**, **4h**, and **4i**. (**a**) Representative images and (**b**) quantitative analysis of migration assays of A549 cells treated with 15 μM compounds **4d**, **4h**, and **4i** for 24, 48, and 72 h. Representative images from three independent experiments are shown (n = 3). Data represent the mean ± SD. *p < 0.05; **p < 0.01; ***p < 0.001; NS, no significant difference compared with DMSO-treated A549 cells.

Wound healing assays are a commonly used method to evaluate cell migration ability^23^. The wound healing assay was performed by treating A549 cells with 15 μM compounds **4d**, **4h**, and **4i** (Fig. 5). Cell migration was inhibited by treatment with compounds **4h** and **4i** in a time-dependent manner but was unaffected by compounds **4d** treatment.

**Figure 6:**
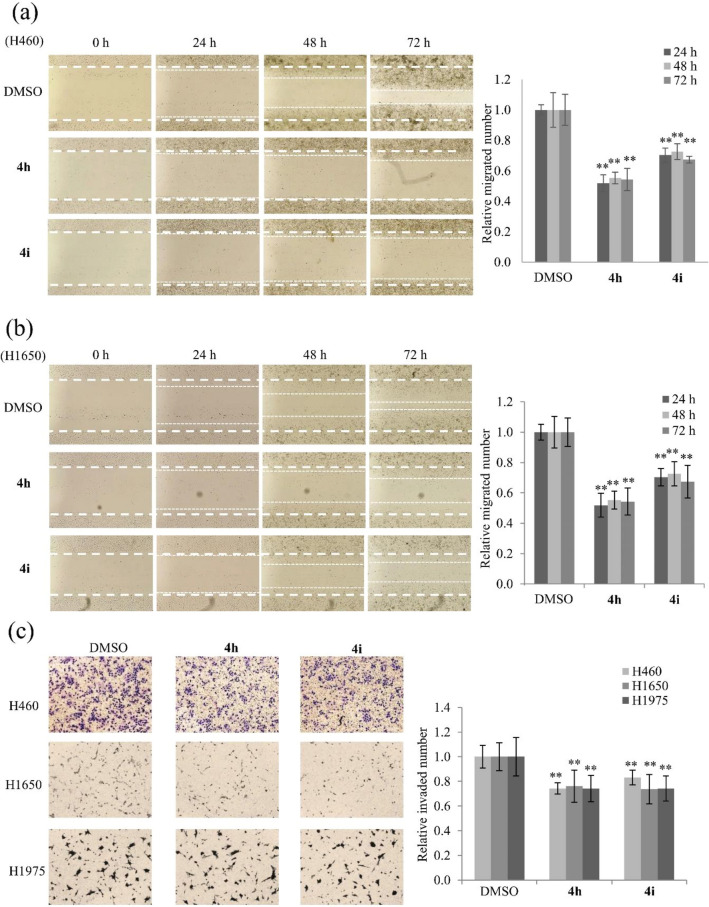
Inhibition of migration and invasion migration of different lung cancer cell lines by compounds **4h** and **4i**. (**a**,**b**) Representative images and quantitative analysis of migration assays of H460, and H1650 cells treated with 15 μM of compounds **4h** and **4i** for 24, 48, and 72 h. (**c**) Representative images and quantitative analysis of invasion assays of H460, H1650, and H1975 cells treated with 15 μM of compounds **4h** and **4i** for each cell line. Representative images from three independent experiments are shown (n = 3). Data represent the mean ± SD. *p < 0.05, **p < 0.01 compared with DMSO-treated cells.

Moreover, the migration assay was performed using the other lung cancer cell lines H460, and H1650. Treatment with compounds **4h** and **4i** significantly decreased abilities of migrated cells in both cell lines (Fig. 6a,b). However, cell migration abilities of H460 and H1650 were also unaffected by treatment with compound **4d** (Supplementary Fig. S2), indicating that it might not be a selection against lung cancer motility. The invasion assays were performed using H460, H1650, and H1975 cells. Treatment with compounds **4h** and **4i** significantly decreased numbers of invaded cells of all three cell lines by more than 25% (*p* < 0.005) (Fig. 6c). Taken together, these results indicate that compounds **4h** and **4i** significantly suppress lung cancer cell motility.

**Figure 7:**
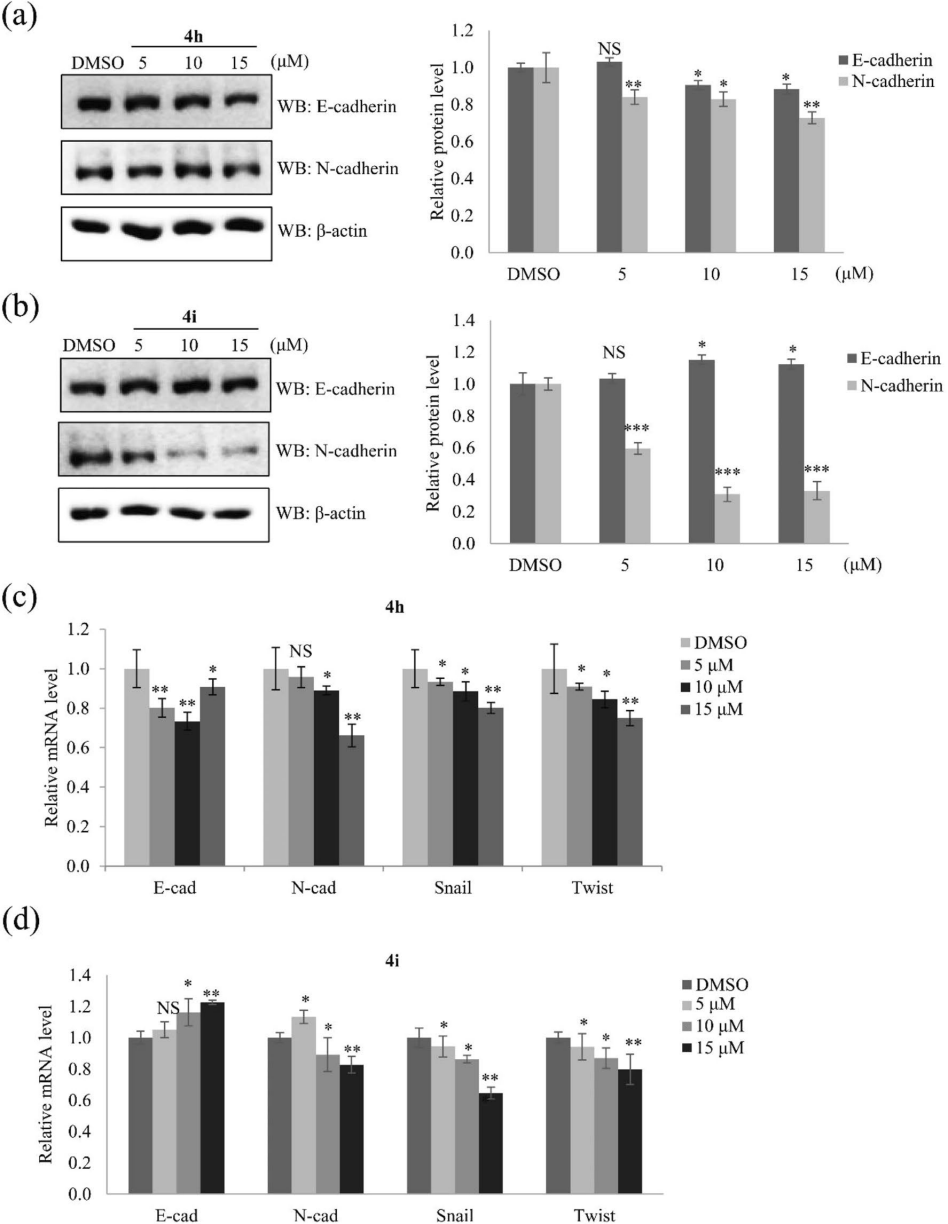
Compounds **4h** and **4i** suppress A549 cell motility by modulating expression of EMT markers and their regulators. (**a**,**b**) Western blotting and quantification of the relative protein levels of E-cadherin and N-cadherin in A549 cells treated with nontoxic concentrations (5, 10, and 15 μM) of compounds **4h** and **4i**. β-Actin served as a loading control. (**c**,**d**) Relative mRNA levels of EMT effectors (E-cadherin and N-cadherin) and their transcription factors (Snail and Twist) in A549 cells treated with nontoxic concentrations (5, 10, and 15 μM) of compounds **4h** and **4i**. The mRNA levels were normalized against that of the housekeeping gene GAPDH. Quantitative data were obtained from at least three independent experiments. Data represent the mean ± SD. Statistical analysis was performed by a one-way analysis of variance. *p < 0.05; **p < 0.01; ***p < 0.001; NS, no significant difference compared with DMSO-treated A549 cells.

The EMT plays a role in lung cancer cell motility and metastasis^24^. In the EMT process, epithelial cells lose cell–cell adhesion and decrease the expression of epithelial cells marker such as E-cadherin, inversely, the expression of mesenchymal cell markers such as N-cadherin is increased, associated with invasive phenotype^25^. Several reports have proved that loss of E-cadherin expression or induction of N-cadherin led to poor prognosis in lung cancer^26–28^. E-cadherin and N-cadherin, as well as their regulators Snail and Twist, are indicators of the EMT^24^. The protein and mRNA expression levels of these EMT markers and their regulators were examined to determine how compounds **4h** and **4i** suppress lung cancer cell motility. Treatment with 15 μM compounds **4h** decreased expression of not only N-cadherin but also E-cadherin and suppressed mRNA expression of Snail and Twist (Fig. 7a,c). Treatment with compound **4i** significantly increased the expression of E-cadherin and downregulated the expression of N-cadherin, Snail, and Twist at a dose-dependent manner (Fig. 7b,d). These results indicate that compound **4i** inhibits expression of EMT markers more than compound **4h**. However, compound **4d** did not affect E/N-cadherin expression as shown in Supplementary Fig. S2c. Taken together, our data demonstrate that compounds **4h** and **4i** inhibit lung cancer cell motility by downregulating EMT markers.

## Conclusion

Ten amidocoumarin derivatives (**4a–4j**) were newly synthesized by a chemical reaction, and their inhibitory effects on lung carcinoma were evaluated. Compounds **4h** and **4i** inhibited invasion and migration of lung cancer cells by modulating expression of EMT effectors. Moreover, the inhibition of EMT markers by compound **4i** was highest, suggesting that it is a potential inhibitor of lung cancer motility. A further biological study is required to elucidate the detailed underlying molecular mechanism.

## Methodology

### Chemistry

#### Synthesis of 4-hydroxy-3-nitrocoumarin (2)

Commercially available 4-hydroxycoumarin (**1**, 3.24 g, 20 mmol, Aldrich) was dissolved in AcOH (60 mL). One portion of sodium nitrite (14 mg, 0.2 mmol) was added to this solution before HNO_3_ (3 mL) dropwise. The reaction was allowed to stir 10 min at room temperature, followed by heating at 60 °C for 1 h in an oil bath. The brown solution was allowed to attain to the room temperature and the pure compound crystallized out from the solution. The suspension was filtered and washed with hexane, and then dried to afford 3-nitro-4-hydroxycoumarin (**2**) as off-yellow crystals. 3.5 g; yield 85%.

#### Synthesis of 3-amino-4-hydroxycoumarin (3)

4-Hydroxy-3-nitrocoumarin (**2**, 3.1 g, 15 mmol) was dissolved in EtOH, and a catalytic amount of Pd/C (30 mg) was added to the mixture at room temperature. The resulting solution was stirred at room temperature under H_2_ atmosphere for 5 h. After completion of the reaction, the mixture was filtered to remove the catalyst with celite pad, washed with MeOH and ethyl acetate, and concentrated to afford an off-white solid. 2.3 g; yield 87%.

#### General procedure for the preparation of 3-aminocoumarins (4a–4j)

3-Amino-4-hydroxycoumarin (**3**, 90 mg, 0.5 mmol) was dissolved in CH_2_Cl_2_. Pyridine (120 uL, 1.5 mmol) was then added, and the mixture was cooled to 0 °C. Variously substituted acid chloride (**4a**–**4j**) was added dropwise at 0 °C, and the mixture was stirred overnight at room temperature. The reaction mixture was evaporated and purified by column chromatography (Hexane/EtOAc, 9:1) to give the compounds 3-aminocoumarins (**4a**–**4j**). The yield of 10 compounds is over 80%. NMR and LC–MS spectra were obtained using a JEOL 400 MHz NMR spectrometer (JEOL, Ltd., Tokyo, Japan) and LC–MS spectrometer (Agilent Technologies, Santa Clara, CA, USA), respectively (Supplementary Fig. S5).

Compd. **4a**: 2-ethoxy-*N*-(4-hydroxy-2-oxo-2*H*-chromen-3-yl)benzamide. ^**1**^**H NMR** (400 MHz, CDCl_3_) δ: 11.06 (s, NH), 8.27 (d, *J* = 7.6 Hz, 1H), 8.04 (d, *J* = 7.6 Hz, 1H), 7.569–7.50 (m, 2H), 7.35–7.30 (m, 2H), 7.13–7.05 (m, 2H), 4.37–4.32 (m, 2H), 1.72–1.68 (m, 3H). ^**13**^**C NMR** (100 MHz, CDCl_3_) δ: 165.35, 161.11, 157.80, 153.41, 150.79, 134.73, 132.64, 131.52, 124.54, 121.33, 118.70, 117.56, 116.22, 112.46, 105.57, 65.81, 14.73. LC–MS (ESI+): Calc. for C_18_H_15_NO_5_ [M + H]^+^: 326.10, Found: 326.2.

Compd. **4b**: *N*-(4-hydroxy-2-oxo-2*H*-chromen-3-yl)-4-methoxybenzamide. ^**1**^**H NMR** (400 MHz, CDCl_3_) δ: 8.80 (s, NH), 8.03 (d, *J* = 6.8 Hz, 1H), 7.94 (d, *J* = 6.8 Hz, 1H), 7.55–7.53 (t, 2H), 7.38–7.33 (t, 2H), 7.01–6.99 (d, J = 6.8 Hz, 2H), 3.90 (s, 3H). ^**13**^**C NMR** (100 MHz, CDCl_3_) δ: 166.98, 163.75, 161.41, 152.72, 150.54, 131.71, 129.78, 124.80, 124.52, 123.60, 117.33, 116.34, 114.42, 104.95, 55.70. LC–MS (ESI+): Calc. for C_17_H_14_NO_5_ [M + H]^+^: 312.09, Found: 312.2.

Compd. **4c**: 2-chloro-*N*-(4-hydroxy-2-oxo-2*H*-chromen-3-yl)benzamide. ^**1**^**H NMR** (400 MHz, CDCl_3_) δ: 9.14 (s, NH), 8.05 (d, *J* = 8.4 Hz, 1H), 7.87 (d, *J* = 8.4 Hz, 1H), 7.59–7.54 (t, 1H), 7.53–7.47 (m, 2H), 7.44–7.40 (m, 1H), 7.38–7.34 (m, 2H). ^**13**^**C NMR** (100 MHz, CDCl_3_) δ: 166.57, 162.75, 160.98, 150.75, 133.08, 132.05, 131.91, 131.64, 131.06, 130.94, 127.51, 124.86, 124.62, 117.07, 116.40, 104.91. LC–MS (ESI+): Calc. for C_16_H_11_ClNO_4_ [M + H]^+^: 316.04, Found: 316.2.

Compd. **4d**: *N*-(4-hydroxy-2-oxo-2*H*-chromen-3-yl)-2-methylbenzamide. ^**1**^**H NMR** (400 MHz, CDCl_3_) δ: 8.50 (s, NH), 8.04 (d, *J* = 8.0 Hz, 1H), 7.62 (d, *J* = 8.4 Hz, 1H), 7.58–7.56 (t, 1H), 7.490–7.30 (m, 4H), 2.61 (s, 3H). ^**13**^**C NMR** (100 MHz, CDCl_3_) δ: 170.52, 161.12, 152.87, 150.65, 137.33, 133.00, 131.90, 131.86, 131.82, 127.66, 126.38, 124.86, 124.55, 117.16, 116.37, 105.08, 20.39. LC–MS (ESI+): Calc. for C_17_H_14_NO_4_ [M + H]^+^: 296.09, Found: 296.2.

Compd. **4e**: *N*-(4-hydroxy-2-oxo-2*H*-chromen-3-yl)-3-methylbenzamide. ^**1**^**H NMR** (400 MHz, CDCl_3_) δ: 8.88 (s, NH), 8.04 (d, *J* = 6.8 Hz, 1H), 7.76–7.74 (m, 2H), 7.58–7.54 (m, 1H), 7.43–7.41 (m, 2H), 7.40–7.341 (m, 2H), 2.45 (s, 3H). ^**13**^**C NMR** (100 MHz, CDCl_3_) δ: 170.60, 167.76, 161.32, 150.61, 139.24, 134.17, 131.86, 131.52, 129.08, 128.20, 124.87, 124.78, 124.75, 116.37, 110.16, 104.86, 21.47. LC–MS (ESI+): Calc. for C_17_H_14_NO_4_ [M + H]^+^: 296.09, Found: 296.2.

Compd. **4f.**: 4-chloro-*N*-(4-hydroxy-2-oxo-2*H*-chromen-3-yl)benzamide. ^**1**^**H NMR** (400 MHz, CDCl_3_) δ: 8.86 (s, NH), 8.04 (d, *J* = 6.4 Hz, 1H), 7.92 (d, *J* = 8.4 Hz, 2H), 7.59–7.55 (t, 1H), 7.53–7.50 (m, 2H), 7.39–7.34 (m, 2H). ^**13**^**C NMR** (100 MHz, CDCl_3_) δ: 166.40, 161.27, 152.96, 150.72, 141.04, 139.93, 132.03, 129.53, 129.07, 124.93, 124.59, 117.11, 116.42, 104.66. LC–MS (ESI+): Calc. for C_16_H_11_ClNO_4_ [M + H]^+^: 316.04, Found: 316.2.

Compd. **4g**: 4-fluoro-*N*-(4-hydroxy-2-oxo-2*H*-chromen-3-yl)benzamide. ^**1**^**H NMR** (400 MHz, CDCl_3_) δ: 9.66 (s, NH), 8.18–8.14 (t, 1H), 8.04 (d, *J* = 7.2 Hz, 1H), 7.64–7.60 (t, 1H), 7.58–7.54 (m, 1H), 7.38–7.33 (m, 2H), 7.26–7.23 (m, 2H). ^**13**^**C NMR** (100 MHz, CDCl_3_) δ: 163.39, 161.00, 153.07, 150.78, 135.35, 135.26, 132.20, 131.93, 125.33, 124.77, 124.59, 117.16, 116.86, 116.62, 116.40, 105.08. LC–MS (ESI+): Calc. for C_16_H_11_FNO_4_ [M + H]^+^: 300.07, Found: 300.2.

Compd. **4h**: 3-chloro-*N*-(4-hydroxy-2-oxo-2*H*-chromen-3-yl)benzamide. ^**1**^**H NMR** (400 MHz, CDCl_3_) δ: 8.87 (s, NH), 8.05 (d, *J* = 8 Hz, 1H), 7.96 (m, 1H), 7.83 (d, *J* = 8 Hz, 1H), 7.61–7.55 (m, 2H), 7.50–7.46 (m, 1H), 7.9–7.35 (m, 2H). ^**13**^**C NMR** (100 MHz, CDCl_3_) δ: 166.15, 161.22, 153.03, 150.65, 135.60, 133.39, 132.08, 130.46, 128.12, 125.49, 124.94, 124.61, 116.43. LC–MS (ESI+): Calc. for C_16_H_11_ClNO_4_ [M + H]^+^: 316.04, Found: 316.2.

Compd. **4i**: *N*-(4-hydroxy-2-oxo-2*H*-chromen-3-yl)-[1,1′-biphenyl]-4-carboxamide. ^**1**^**H NMR** (400 MHz, CDCl_3_) δ: 8.95 (s, NH), 8.05–8.02 (m, 3H), 7.76 (d, *J* = 8.8 Hz, 2H), 7.65 (d, *J* = 8.8 Hz, 2H), 7.59–7.54 (m, 1H), 7.50–7.47 (m, 2H), 7.44–7.35 (m, 3H). ^**13**^**C NMR** (100 MHz, CDCl_3_) δ: 167.19, 161.35, 152.93, 150.61, 146.23, 139.52, 131.88, 130.07, 129.14, 128.58, 128.25, 127.78, 127.38, 124.87, 124.58, 117.24, 116.39, 104.87. LC–MS (ESI+): Calc. for C_22_H_16_NO_4_ [M + H]^+^: 358.11, Found: 358.2.

Compd. **4j**: 2-fluoro-N-(4-hydroxy-2-oxo-2H-chromen-3-yl)benzamide. ^**1**^**H NMR** (400 MHz, CDCl_3_) δ: 8.05–8.01 (m, 2H), 7.60–7.54 (m, 2H), 7.25–7.14 (m, 4H). ^**13**^**C NMR** (100 MHz, CDCl_3_) δ: 169.89, 164.01, 161.41, 135.66, 135.57, 132.83, 124.20, 124.16, 117.87, 117.79, 117.35, 117.12. LC–MS (ESI+): Calc. for C_16_H_11_FNO_4_ [M + H]^+^: 300.07, Found: 300.2.

### Biological evaluation

#### Cell culture and material

The lung cancer cell lines A549, H1650, H1975, and H460 were obtained from the Korean Cell Line Bank (Seoul, South Korea). Cells were cultured in RPMI 1640/DMEM culture medium (Gen Depot, TX, USA) supplemented with 10% fetal bovine serum (Gen Depot, TX, USA) and 1% Penicillin–Streptomycin solution. Cells were cultured in 5% CO_2_ in a humidified atmosphere at 37 °C. 4-Hydroxycoumarin was purchased from Sigma-Aldrich (H23805).

#### MTT assay

Cells (2 × 10^4^ cells/well) were seeded on a 96-well plate, grown overnight, and then treated with 4-hydroxycoumarin and its derivatives **4a**–**4j** at concentrations from 3.125 to 100 μM for 48 h. After incubation with MTT at 37 °C, cells were lysed with 150 μL of DMSO (Sigma-Aldrich, St. Louis, USA) and absorbance was measured spectrophotometrically at 540 nm.

#### Invasion assay

Invasion assays were performed in Transwell chambers (Corning, New York, USA) with 8 μm pore size polycarbonate membrane coated with 1% gelatin. A549, H1650, H1975, and H460 cells were plated at 2 × 10^6^ cells/mL in RPMI1640 containing 0.2% bovine serum albumin (BSA) in the upper compartment of the chamber. The lower chamber was filled with 400 μL RPMI containing 0.2% BSA and 10 μg/mL fibronectin (EMD MilliporeCorp., Billerica, MA, USA) as a chemoattractant. A549 cells were treated with 5 μM of 4-hydroxycoumarin and its derivatives (**4a**–**4j**) for 48 h. H1650, H1975, and H460 cells were treated with 5, 10, and 15 μM of derivatives **4h** and **4i** for 48 h. After incubation, the cells in the upper chamber were fixed with Diff Quik kit (Sysmex, Kobe, Japan). The numbers of cells in five fields per chamber were counted using a Nikon Eclipse 400fluorescence microscope (Nikon Instech, Co., Ltd, Kawasaki, Japan) and iSolution FL Auto × 64 software (IMT iSolution Inc., Vancouver, Quebec, Canada).

#### Wound healing assay

A549, H460, and H1650 cells were plated at a density of 2.5 × 10^5^ cells/well and grown overnight to the confluence. Monolayer cells were scratched to create a wound. The cells were then washed twice and incubated in RPMI1640 culture medium supplemented with 2% FBS with 15 μM of derivatives **4d**, **4h**, and **4i**. For the quantitation of relative migration ability, photographs of cells were taken at 0, 24, 48, and 72 h after wounding to measure the width of the wound. The distance migrated by the cells was calculated as the difference between the edges of the wound at time point 1 and at time point 2. For each sample, an average of five wound distances was taken to determine the average rate of migration at the time-course of 24, 48, and 72 h. The migrating rate was determined with the following formula: migrating rate [%] = (width t1 [mm] − width t2 [mm]) / width t1 × 100%.

#### Western blots

A549 cells were treated with 5, 10, or 15 μM concentrations of compounds **4d**, **4h** and **4i** for 24 h. Cells were lysed and the proteins (25 μg) were separated by 10% polyacrylamide gel electrophoresis containing 0.1% SDS and electrophoretically transferred to nitrocellulose membranes. Anti-E-cadherin (#3195) (Cell signaling) and anti-N-cadherin(#13A9) (Cell signaling) were used as primary antibodies for the detection of EMT markers. Anti-ß-actin (Santa Cruz Biotechnology) was used as internal standards. All results are representative from at least three independent experiments. Bands were measured by Multi Gauge version 3.0 (Fuji photo film Co., Ltd., Tokyo, Japan) and their relative density calculated based on the density of the ß-actin bands in each sample. Values were expressed as arbitrary densitometric units corresponding to signal intensity. Cropped gels retain important bands, but whole gel images with membrane edges visible are available in Supplementary Figs. S3 and S4.

#### qRT-PCR

Briefly, total RNA was isolated from compounds **4h** or **4i** treated A549 cells using RNAiso Plus (TaKaRa, Otsu, Shiga 520-2193, Japan) and then total RNAs (1 μg) were used to synthesize cDNA using a M-MLV reverse transcriptase kit (Invitrogen, Carlsbad, CA, USA) and SYBR green (Enzynomics, Seoul, Korea). qRT-PCR reactions and analysis were performed using CFX (Bio-Rad, Hercules, CA, USA). The primers used for real-time PCR were as follows: E-cadherin (forward) 5′-cagaaagttttccaccaaag-3′ and (reverse) 5′-aaatgtgagcaattctgctt-3′; N-cadherin (forward) 5′-ctcctatgagtggaacaggaacg-3′ and (reverse) 5′-ttggatcaatgtcataatcaagtgctgta-3′; Snail (forward) 5′-tcccgggcaatttaacaatg-3′ and (reverse) 5′-tgggagacacatcggtcga-3′; Twist (forward) 5′-cgggagtccgcagtctta-3′ and (reverse) 5′-tgaatcttgctcagcttgtc-3′; and GAPDH (forward) 5′-atcaccatcttccaggagcga-3′ and (reverse) 5′-agttgtcatggatgaccttggc-3′. Target gene expression was normalized relative to the expression of GAPDH in the same sample.

### Statistical analysis

All in vitro experiments were performed in triplicate. Data are presented as the mean standard deviation (SD) of 3 independent experiments with 3 replicates each. The student's t-test was utilized to determine statistical significance between two groups, and analysis of variance was utilized between three or more groups, respectively. Values of *p* < 0.05 were considered statistically significant. All statistical analyses were performed using Sigma Plot 12.5 software (Systat software, Erkrath, Germany).

## Supplementary Information

Supplementary Figures.

## Data Availability

All data generated or analyzed during this study are included in this published article and its supplementary information file.
