# Supplementary material for: Synthesis of coumarin derivatives and investigation of their inhibitory effects on lung cancer cell motility
Source: Sci Rep. 2022 Dec 14;12:21635. doi: 10.1038/s41598-022-26212-z (PMC9751305; doi:10.1038/s41598-022-26212-z)

**Supplementary Data for**

**Synthesis of coumarin derivatives and investigation of their inhibitory effects on lung cancer cell motility**

**Rui Zhou, Young Hyun Yu, Hangun Kim*, Hyung-Ho Ha***

*Correspondence. Email: [hangunkim@sunchon.ac.kr](mailto:hangunkim@sunchon.ac.kr) (HK); [hhha@sunchon.ac.kr](mailto:hhha@sunchon.ac.kr) (HHH)

**Supplementary Data**

**Supplementary Figure S1. Cell viabilities of H460 and H1975 after treatment with 4-hydroxycoumarin and its derivatives 4a–4j.**

(a) Relative viabilities of H460 and H1975 after treatment with 4-hydroxycoumarin and its derivatives **4a–4j** for 48 h at concentrations ranging from 3.125 to 100 μM measured by the MTT assay. Data represent the mean ± SD. *p < 0.05 compared with dimethyl sulfoxide (DMSO)-treated cells.

**Supplementary Figure S2. The effect of compound 4d on cell migration and EMT markers in lung cancer cells.**

(a,b) Representative images and quantitative analysis of migration assays of H460, and H1650 cells treated with 15 μM of compounds **4d** for 24, 48, and 72 h. Representative images from three independent experiments are shown (n = 3). (c) Western blotting and quantification of the relative protein levels of E-cadherin and N-cadherin in A549 cells treated with nontoxic concentrations (5, 10, and 15 μM) of compounds **4d**. β-Actin served as a loading control. Data represent the mean ± SD. *p < 0.05; NS, no significant difference compared with DMSO-treated cells.

**Supplementary Figure S3. Full-length blots shown in Figure 7.**

**Supplementary Figure S4. Full-length blots shown in supplementary Figure S2.**

**Supplementary Figure S5. ^1^H NMR, ^13^C NMR, and LC-MS data of 4a–4j.**


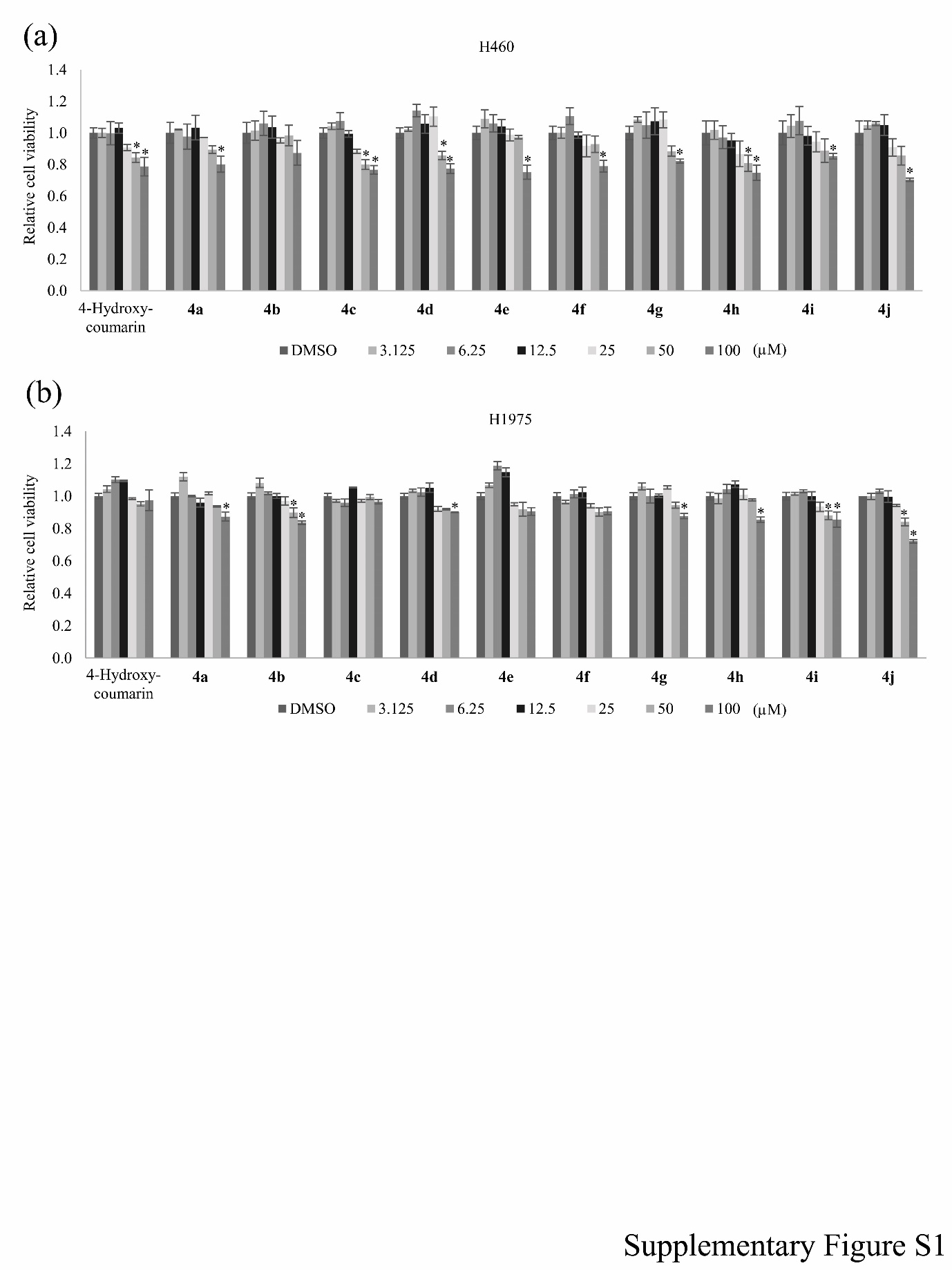

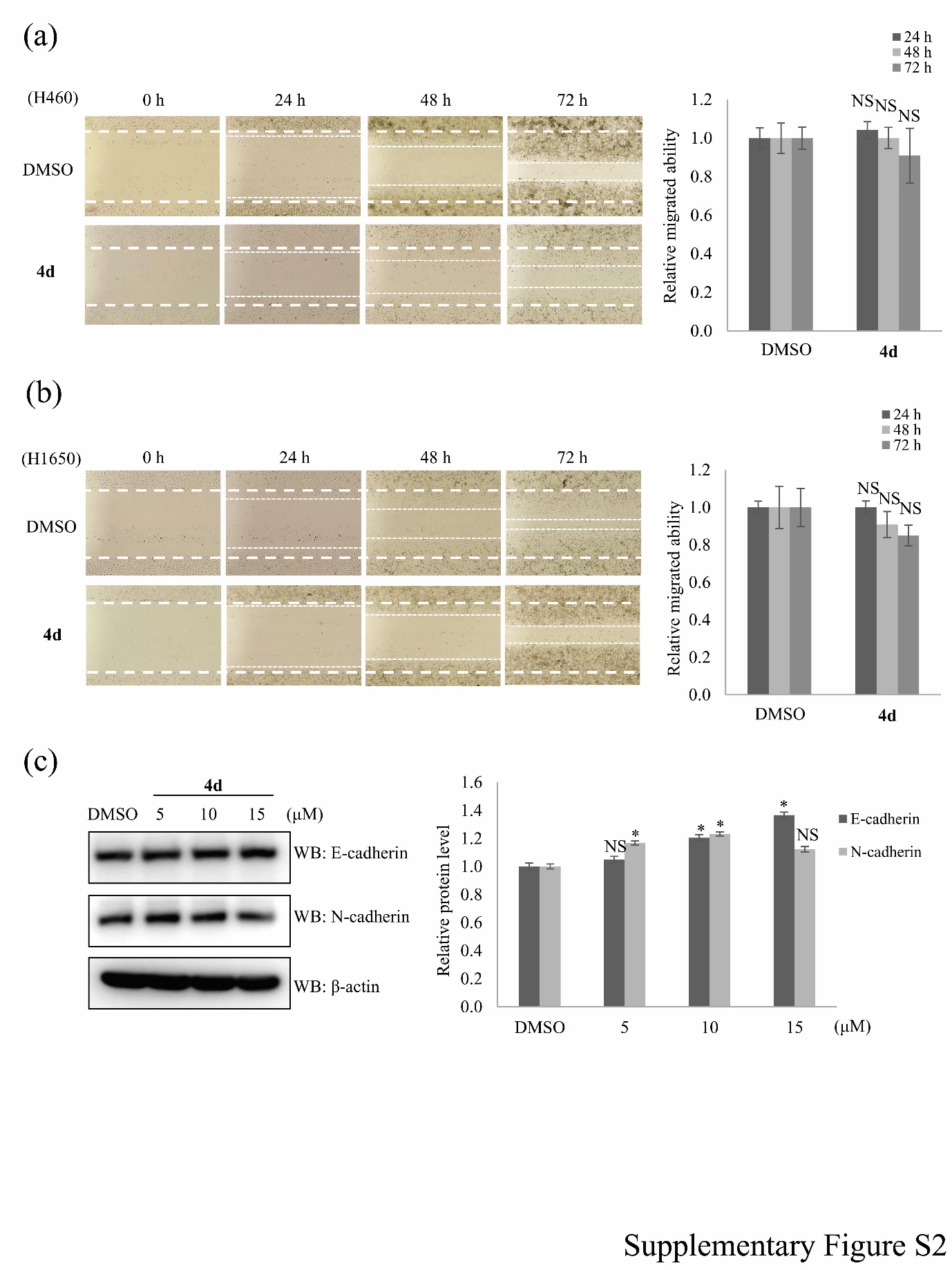

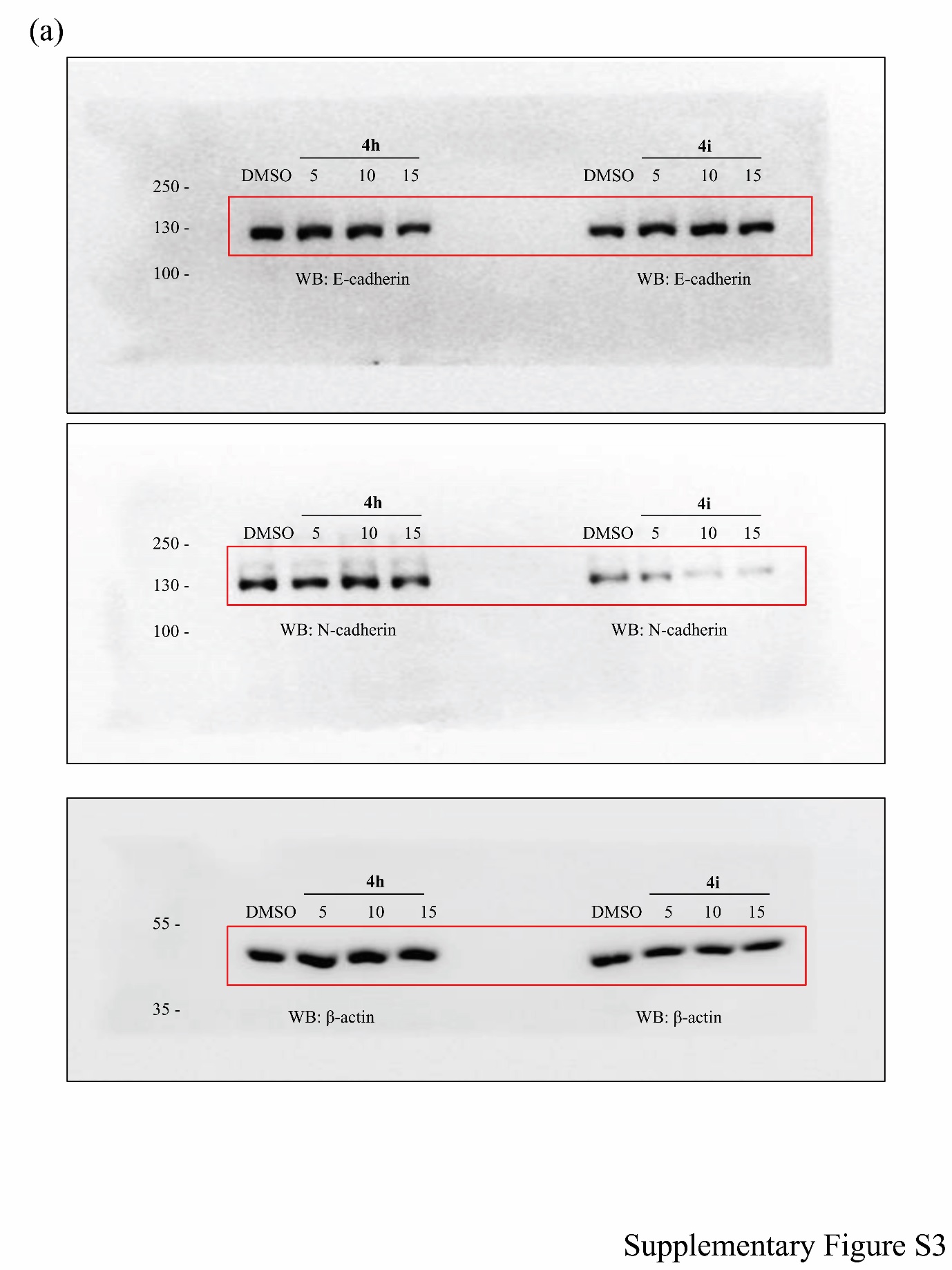

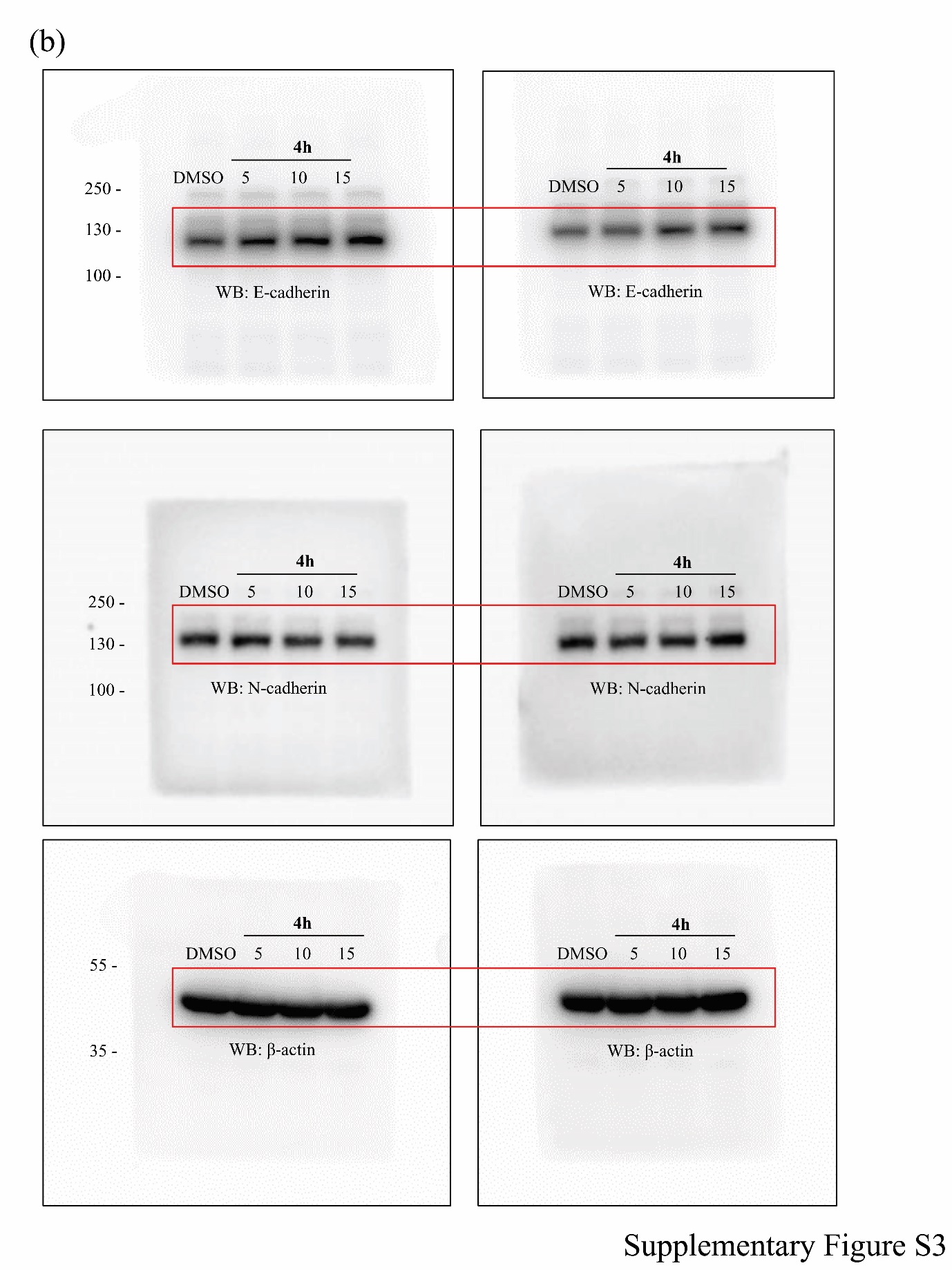

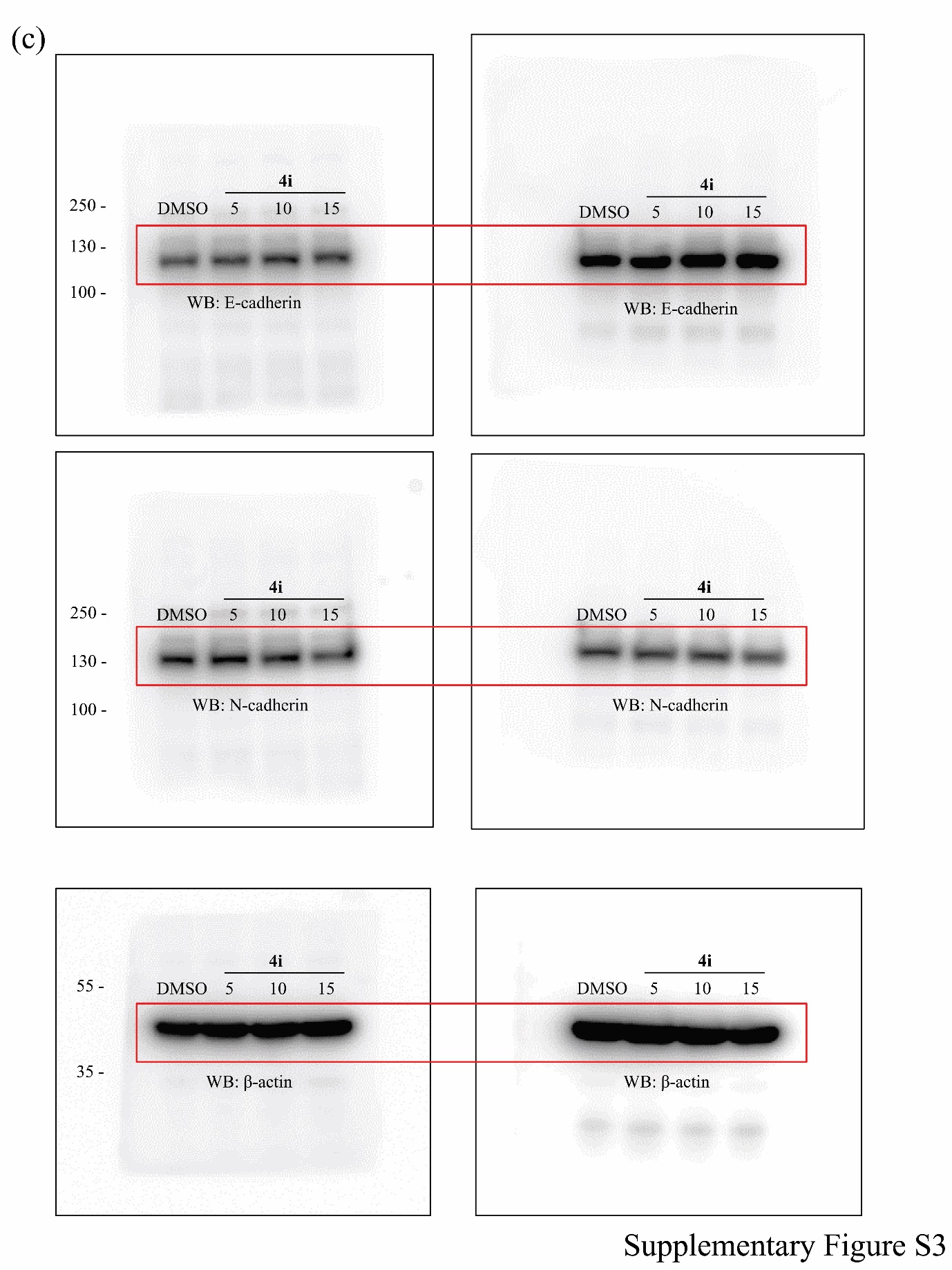

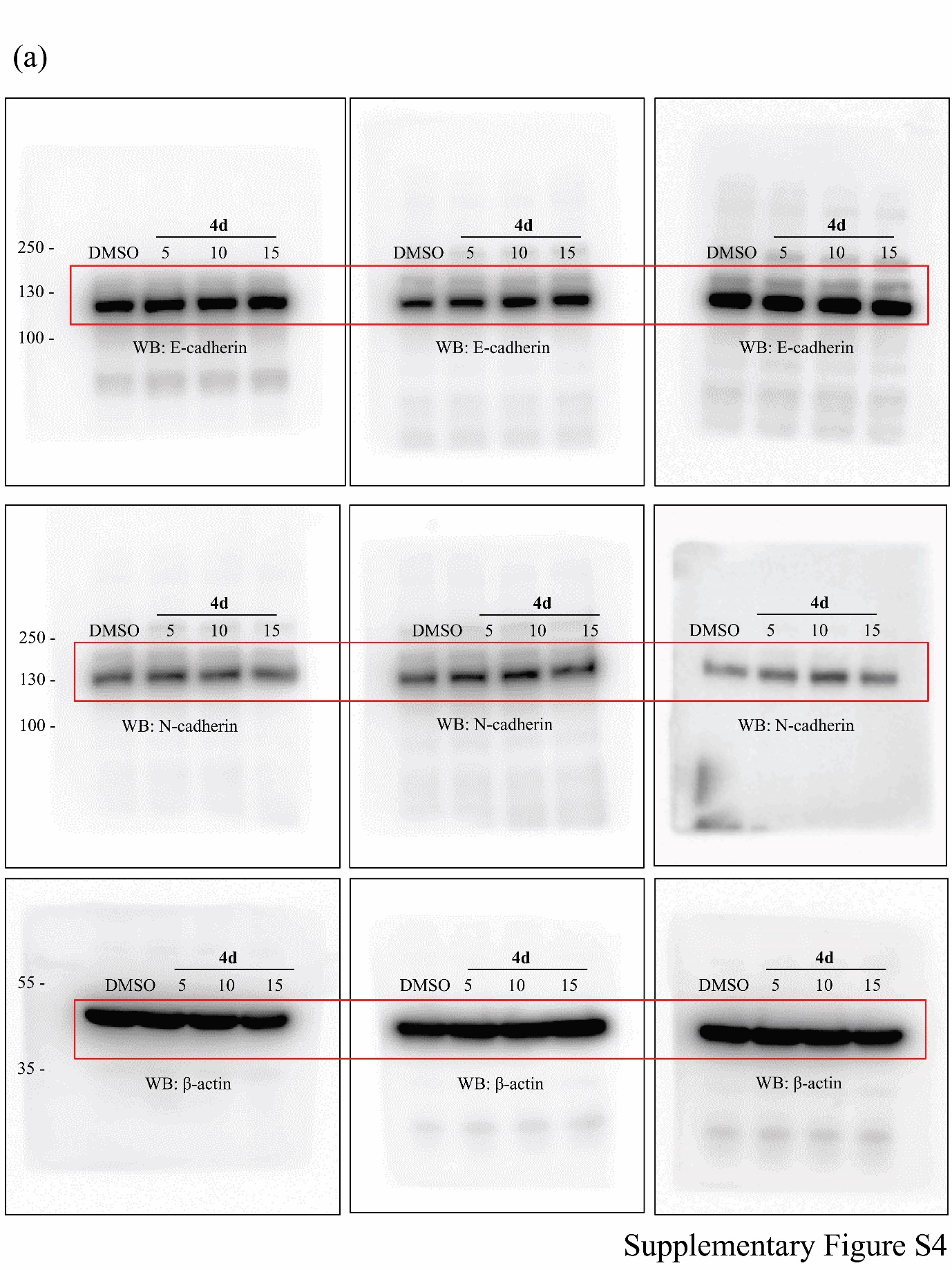


Supplementary Figure S5 (a)

**Compd. 4a: 2-ethoxy-N-(4-hydroxy-2-oxo-2H-chromen-3-yl)benzamide : ^1^H & ^13^C NMR**


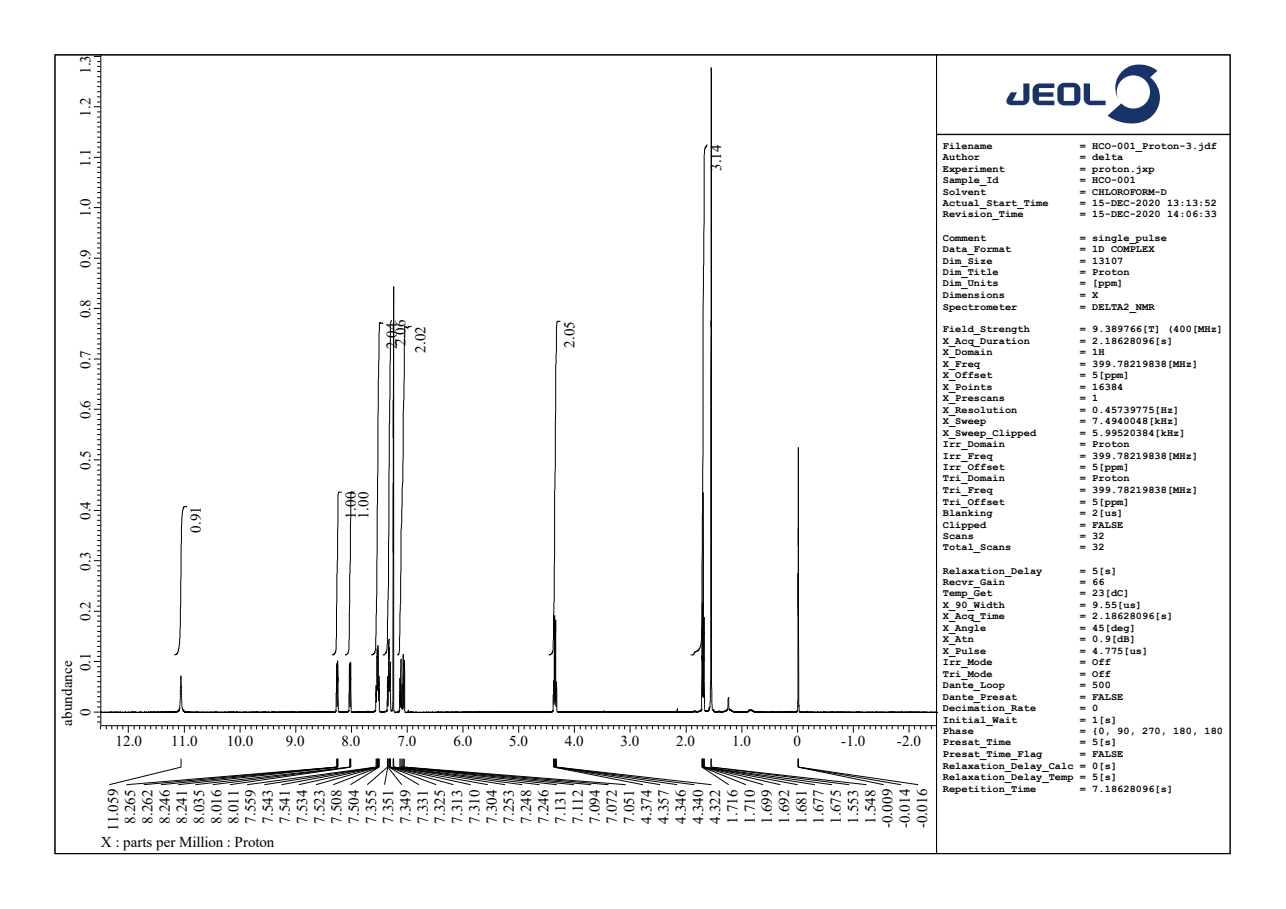


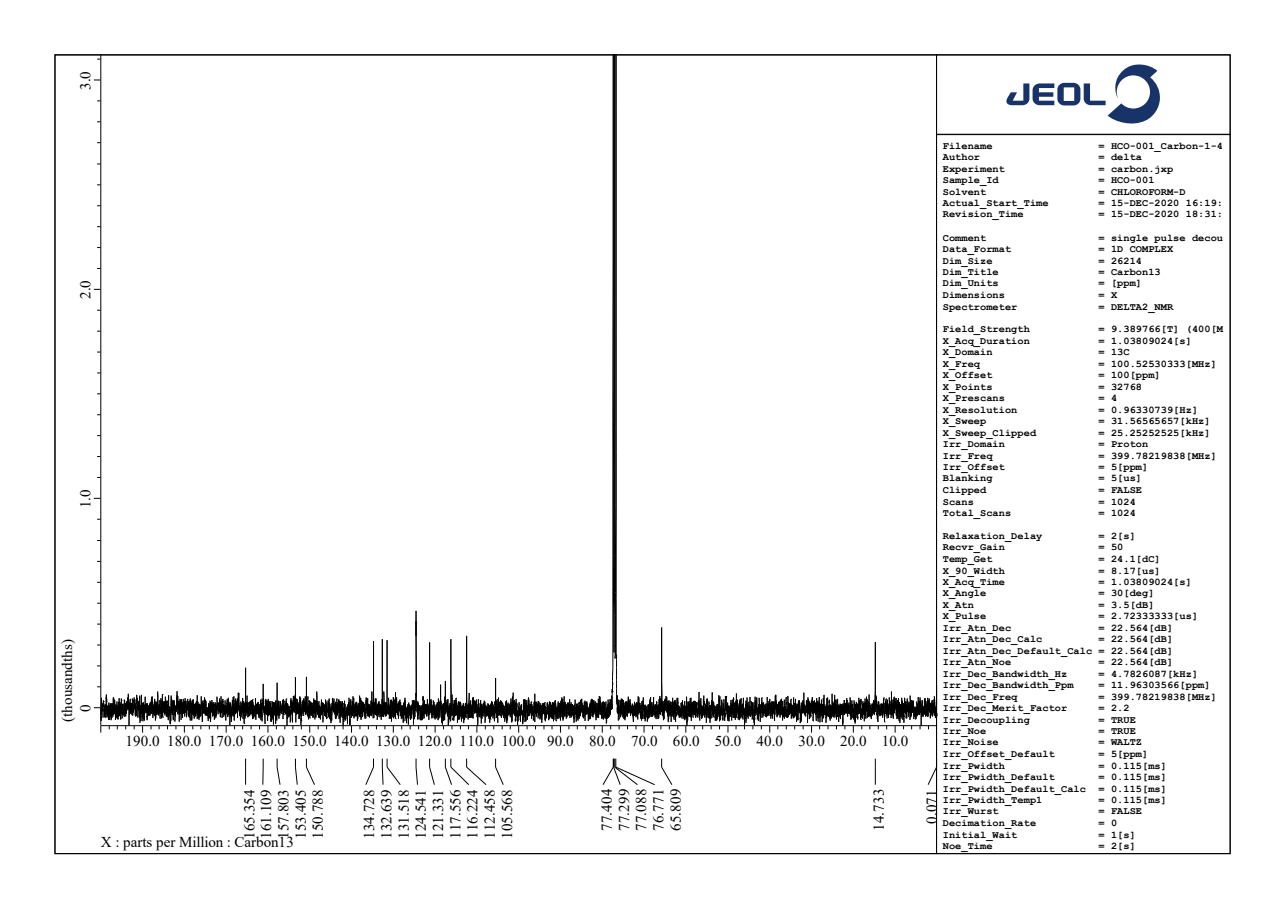


Supplementary Figure S5 (b)

**Compd. 4a: 2-ethoxy-N-(4-hydroxy-2-oxo-2H-chromen-3-yl)benzamide: LC-MS Data**

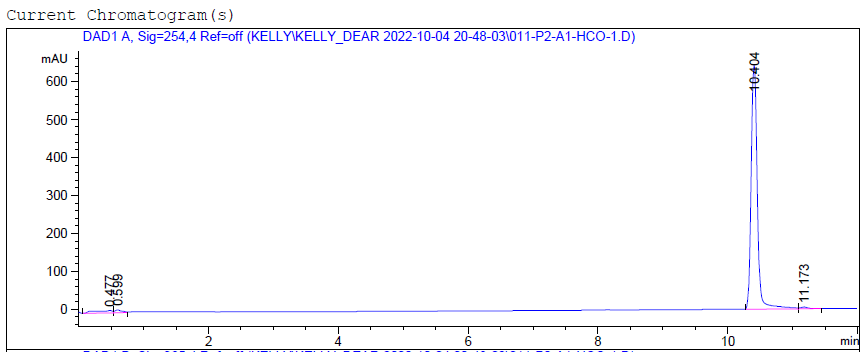


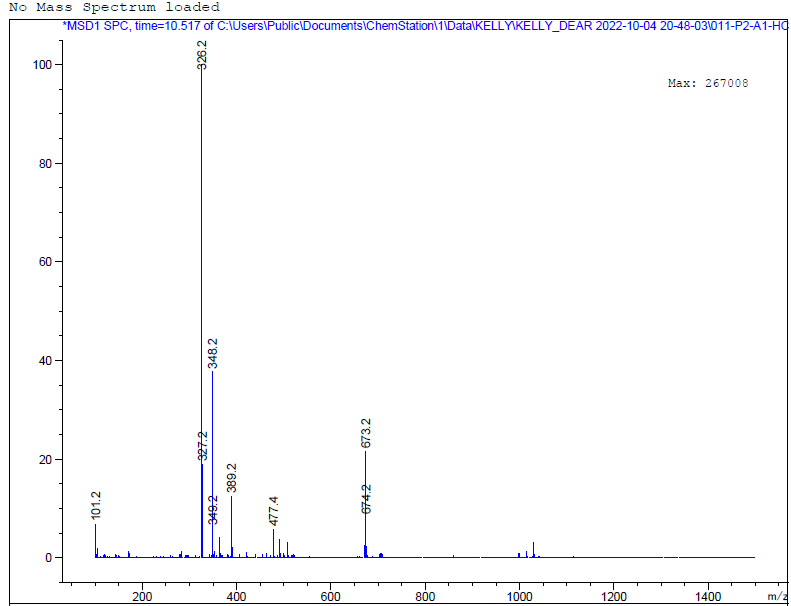


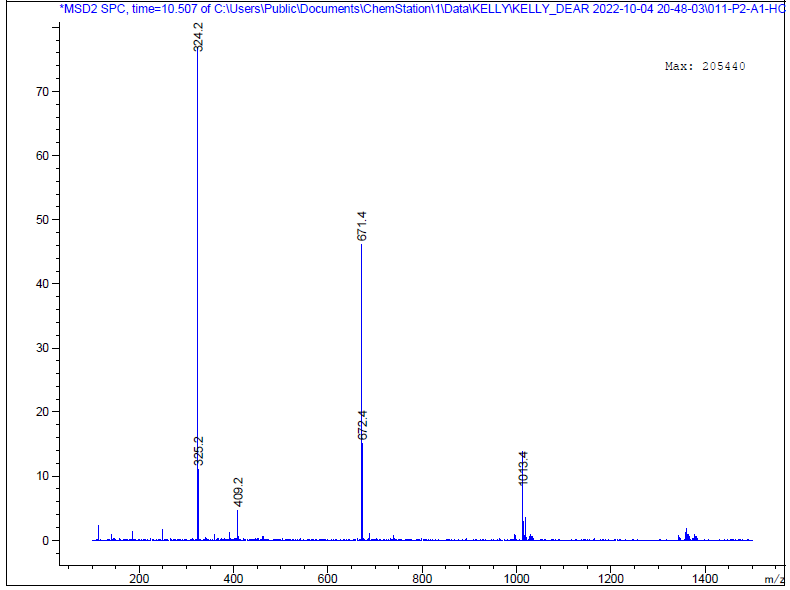


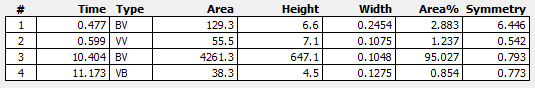


Supplementary Figure S5 (c)

**Compd. 4b: N-(4-hydroxy-2-oxo-2H-chromen-3-yl)-4-methoxybenzamide : ^1^H & ^13^C NMR**


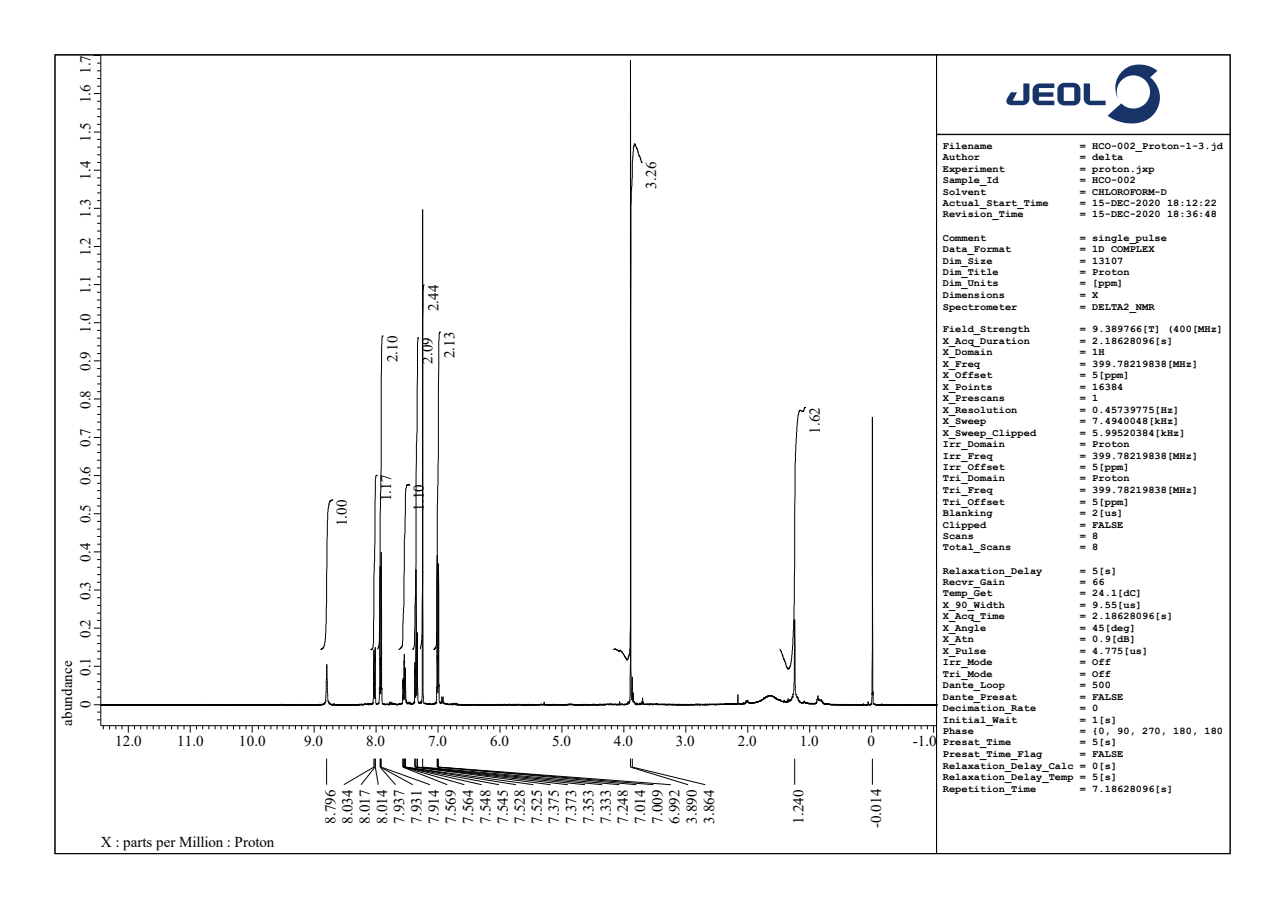

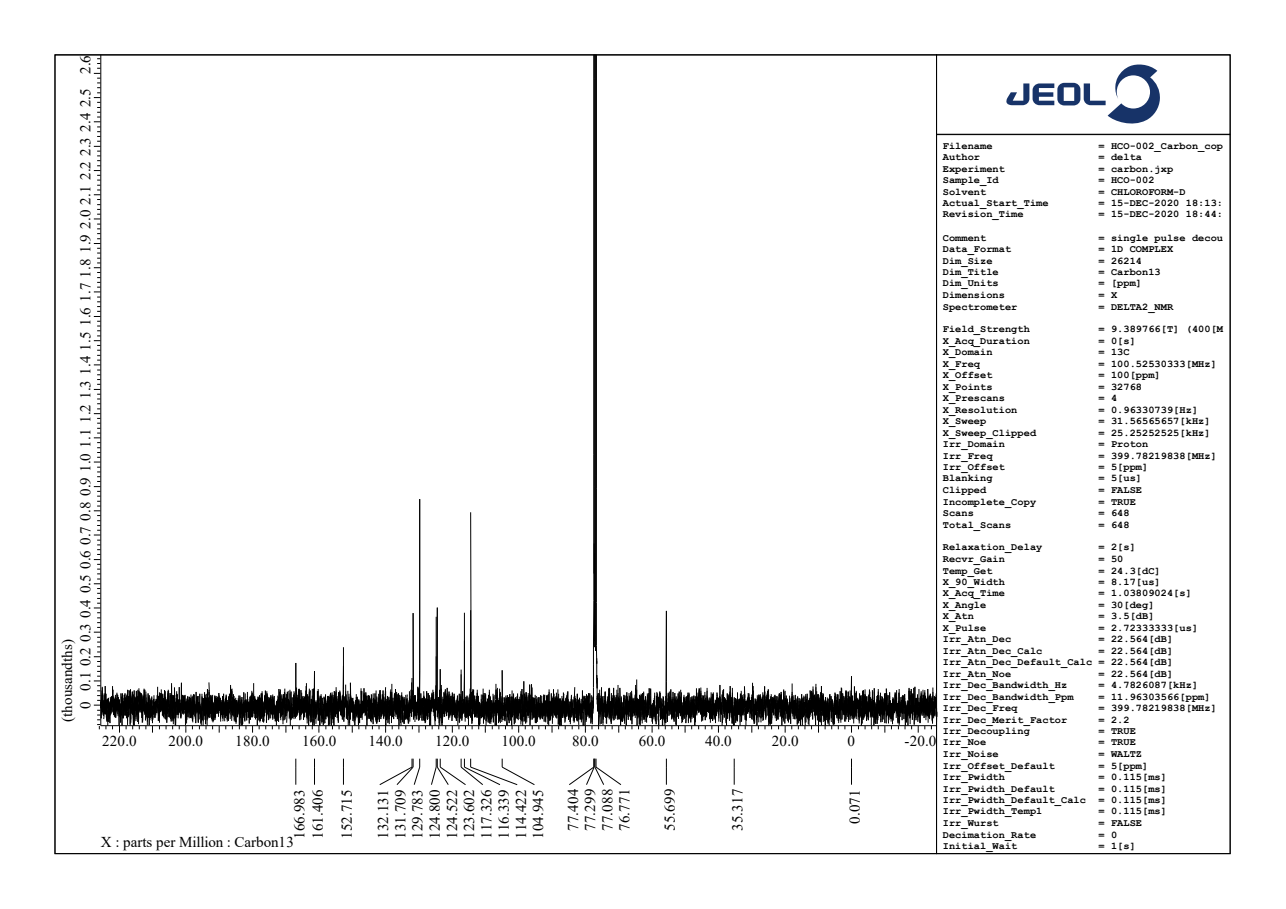


Supplementary Figure S5 (d)

**Compd. 4b: N-(4-hydroxy-2-oxo-2H-chromen-3-yl)-4-methoxybenzamide: LC-MS Data**

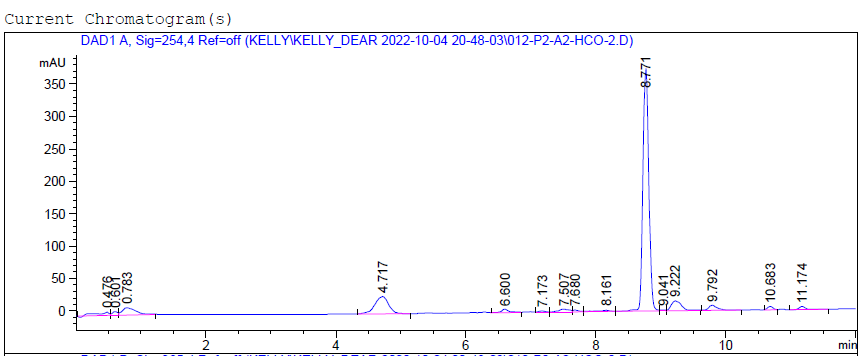


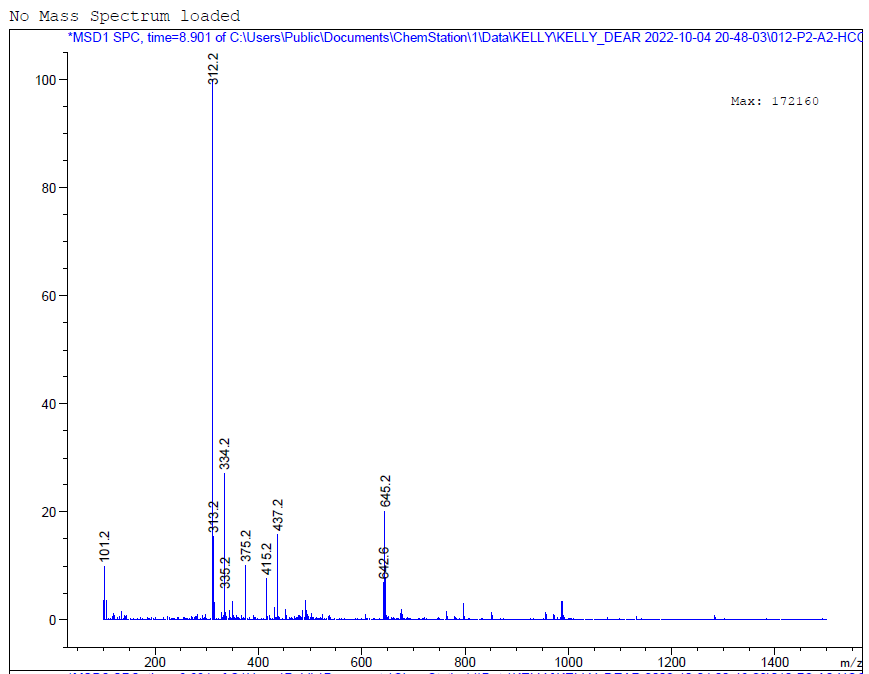


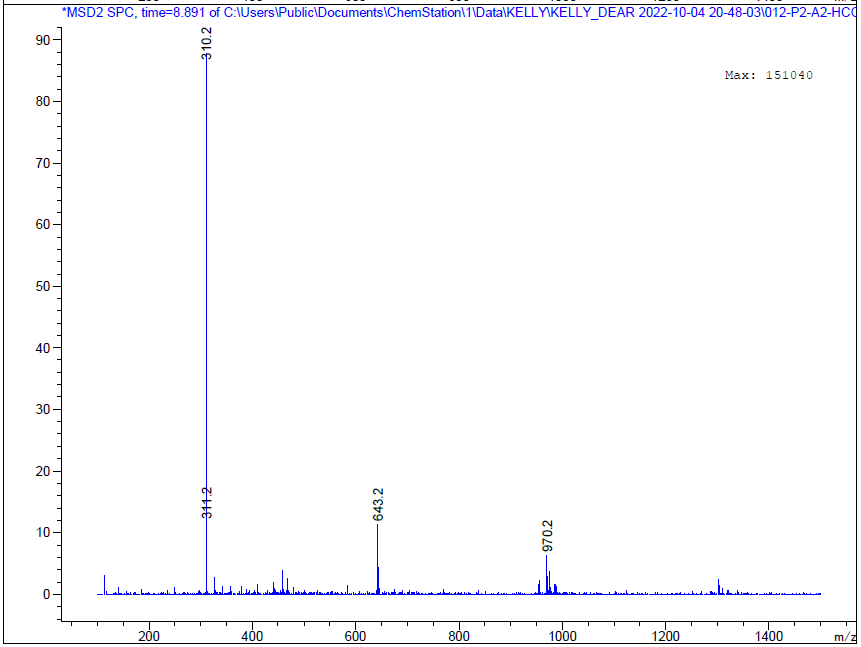


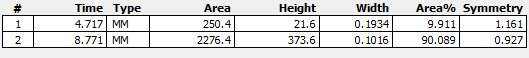


Supplementary Figure S5 (e)

**Compd. 4c: 2-chloro-N-(4-hydroxy-2-oxo-2H-chromen-3-yl)benzamide: ^1^H & ^13^C NMR**


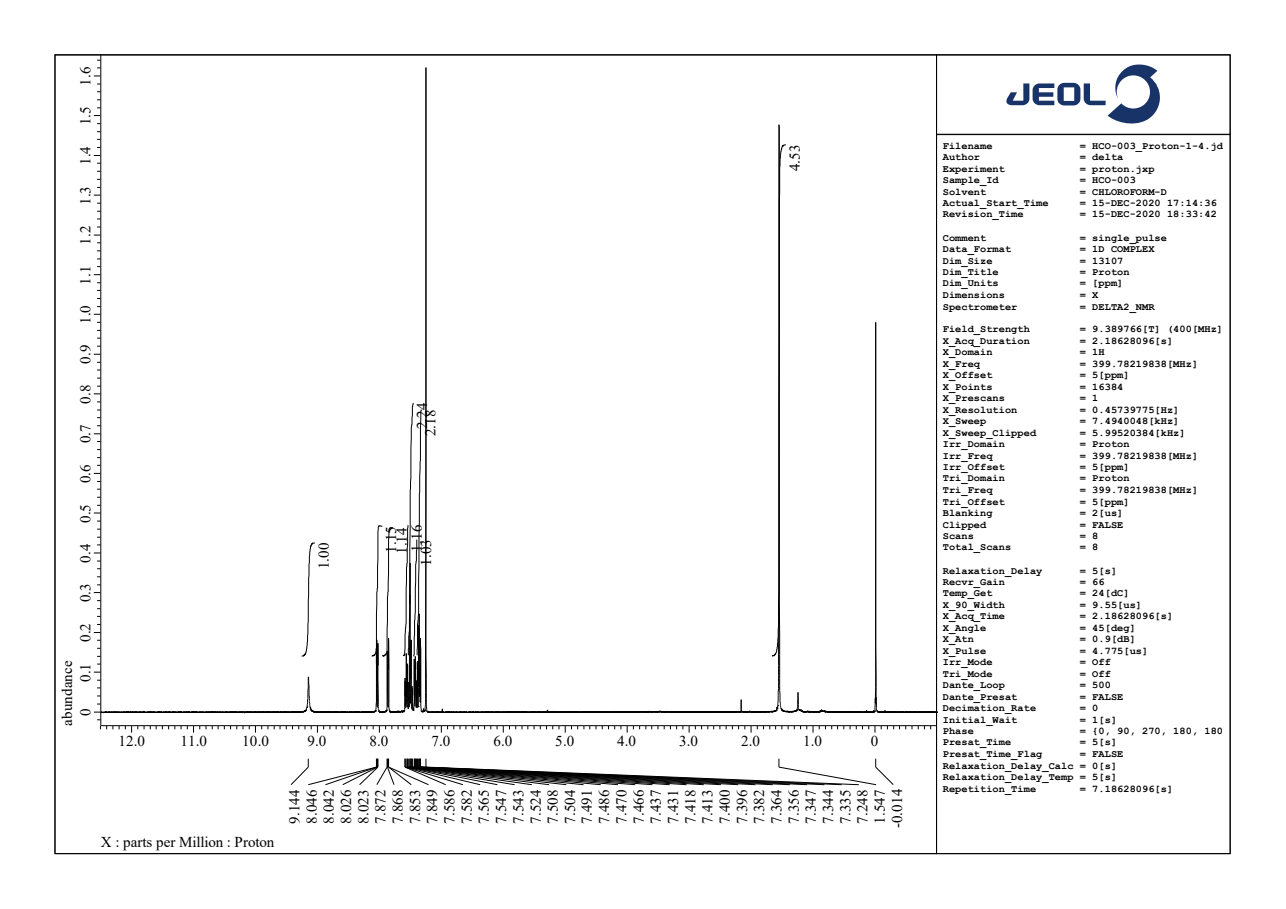

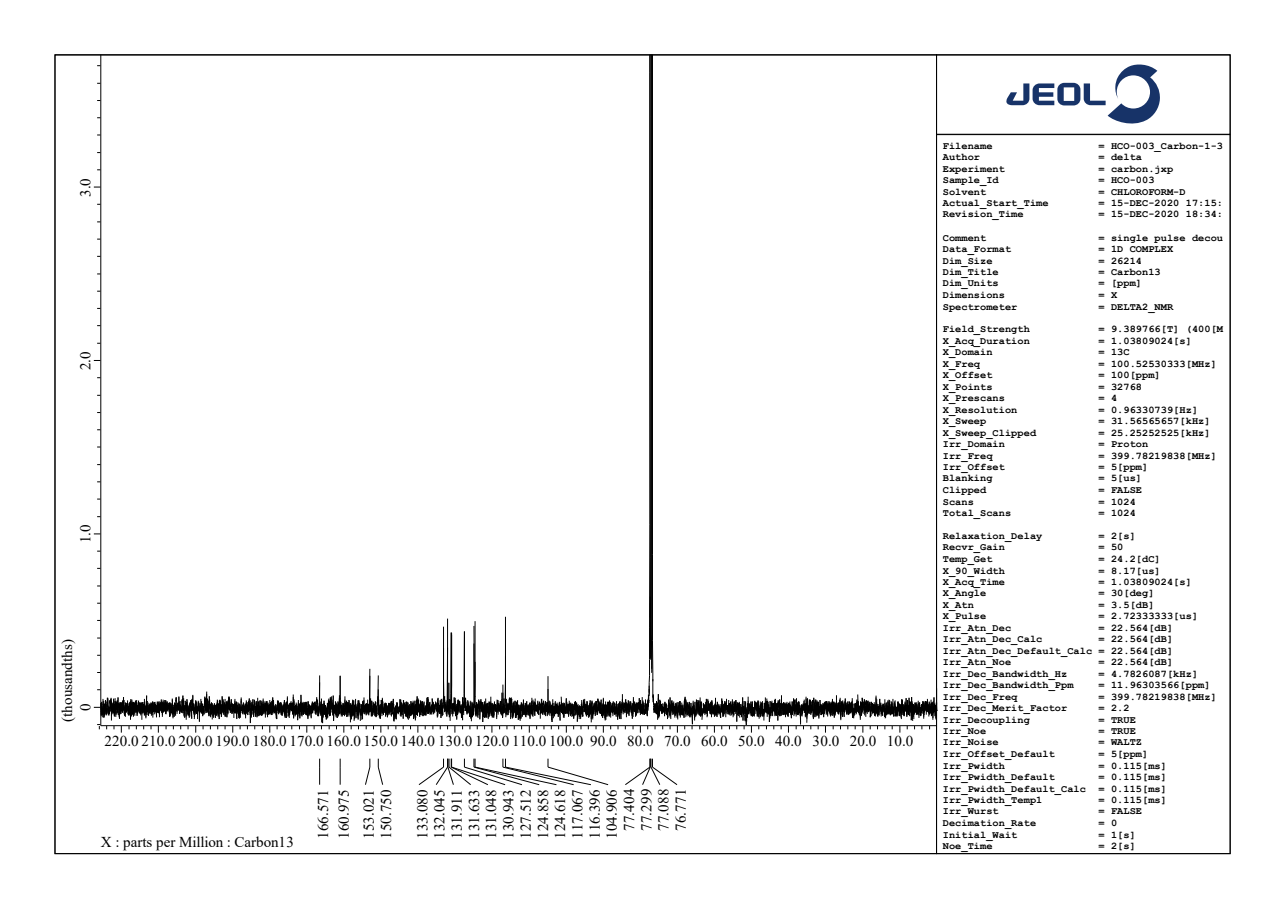


Supplementary Figure S5 (f)

**Compd. 4c: 2-chloro-N-(4-hydroxy-2-oxo-2H-chromen-3-yl)benzamide: LC-MS Data**

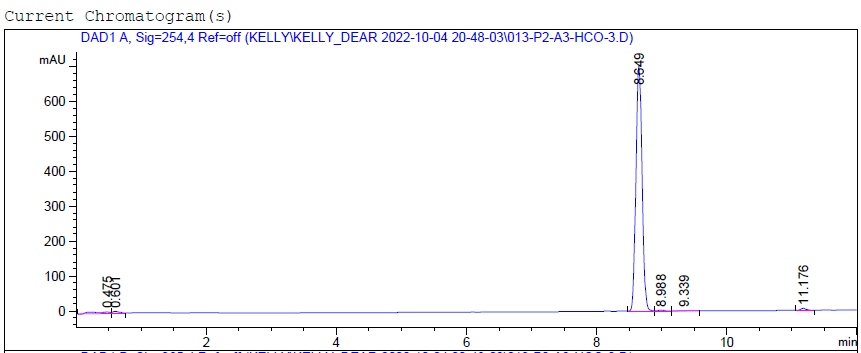


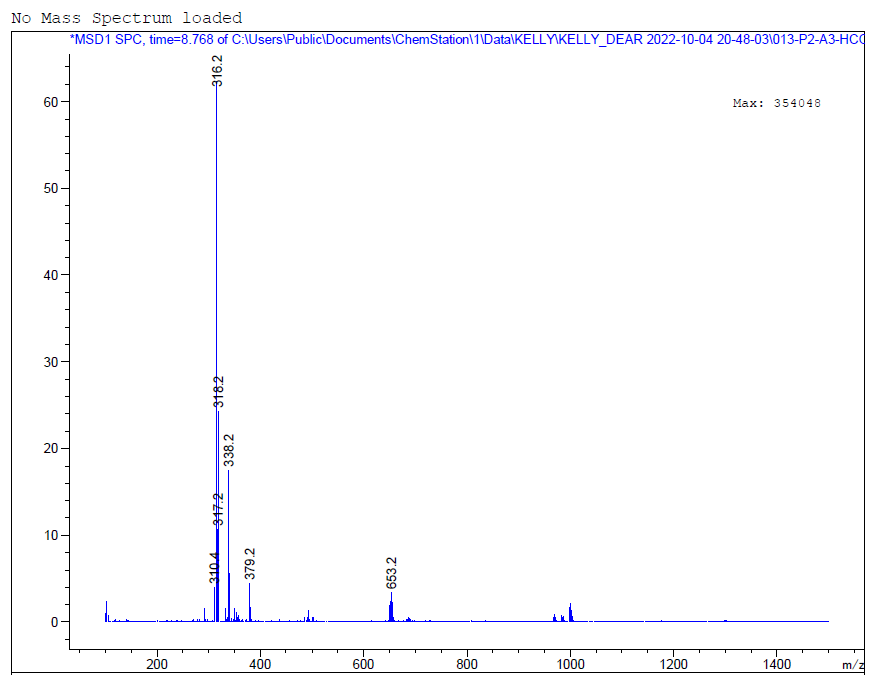


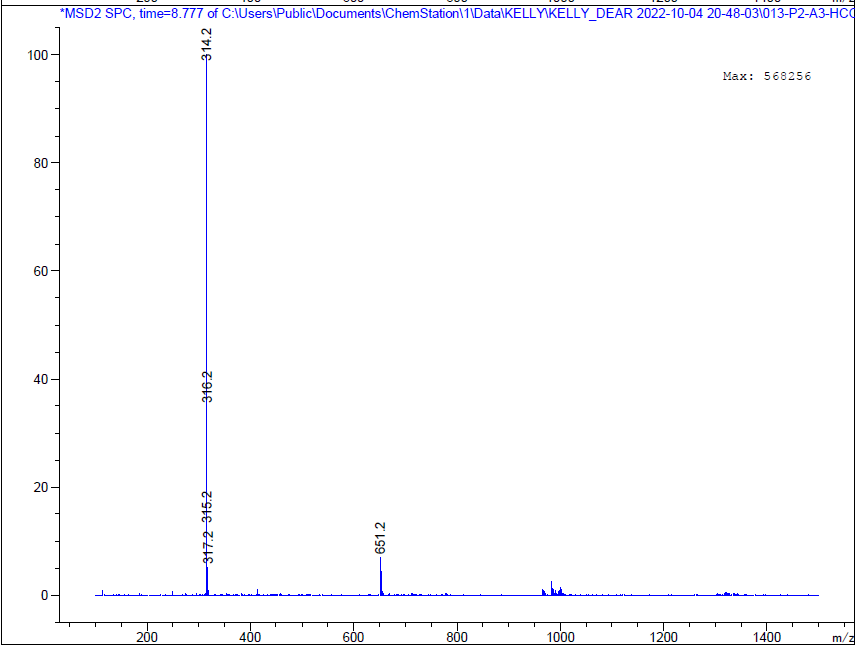


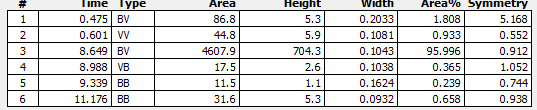


Supplementary Figure S5 (g)

**Compd. 4d: N-(4-hydroxy-2-oxo-2H-chromen-3-yl)-2-methylbenzamide: ^1^H & ^13^C NMR**


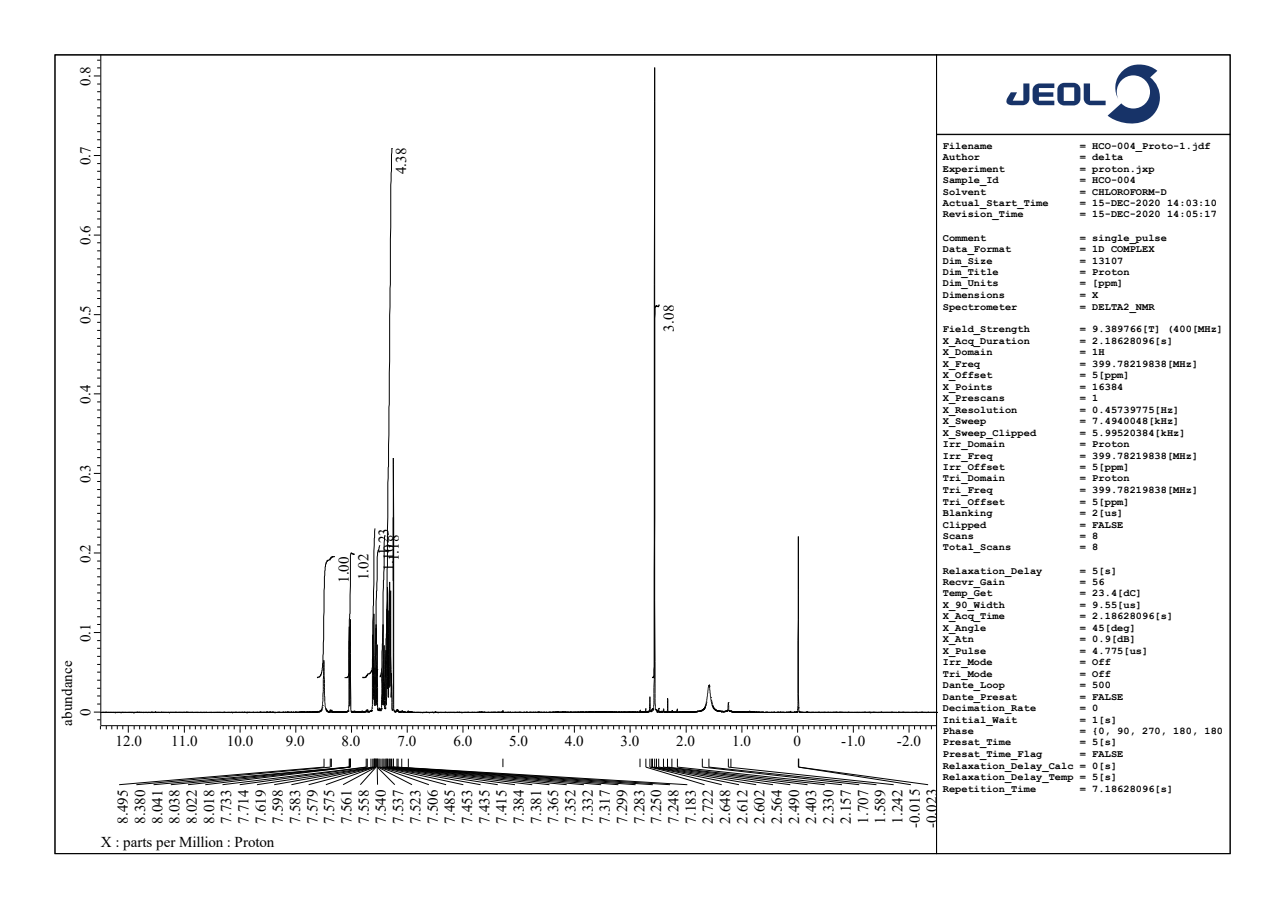

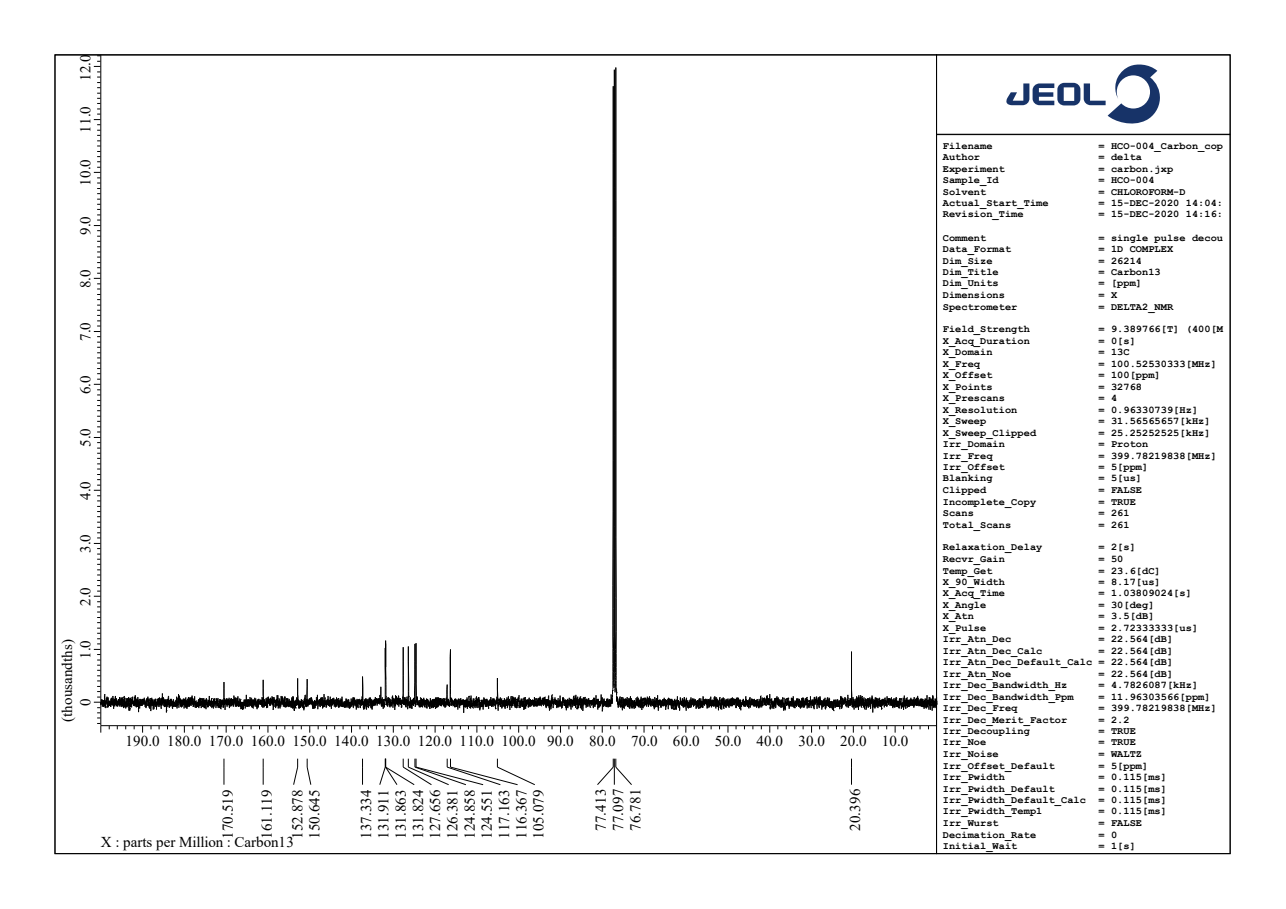


Supplementary Figure S5 (h)

**Compd. 4d: N-(4-hydroxy-2-oxo-2H-chromen-3-yl)-2-methylbenzamide: LC-MS Data**

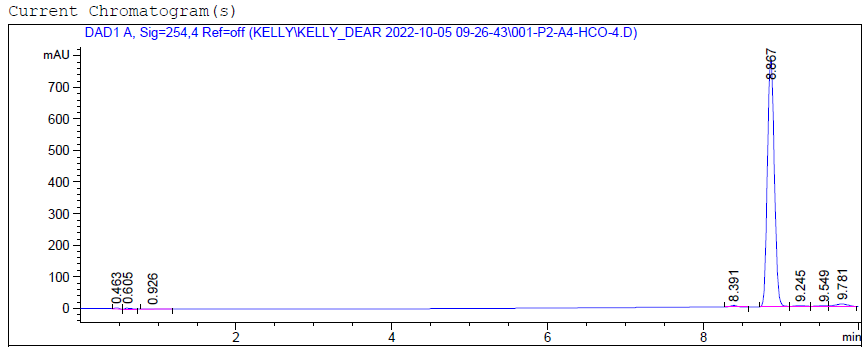


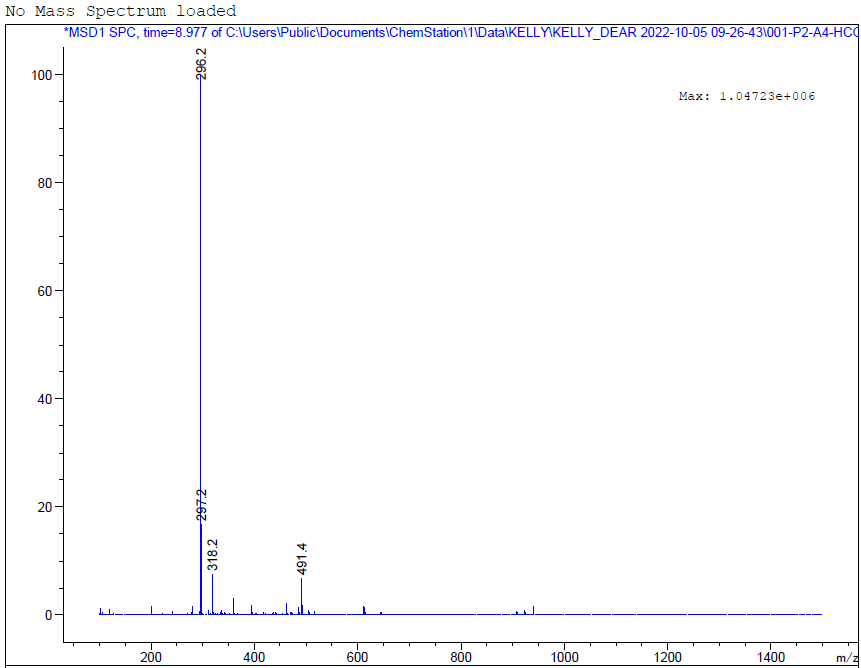


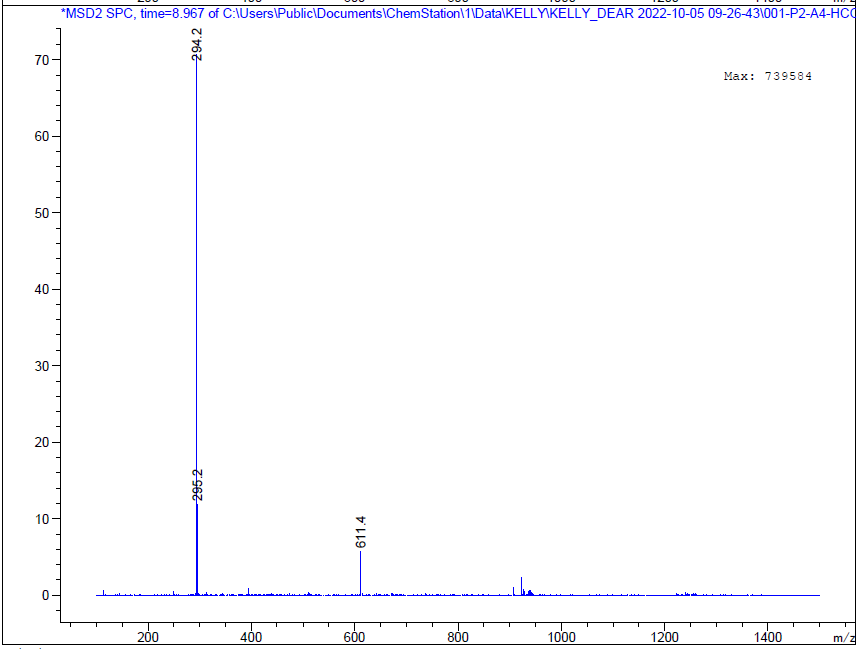


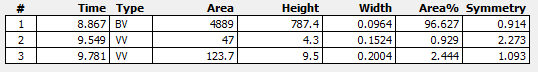


Supplementary Figure S5 (i)

**Compd. 4e: N-(4-hydroxy-2-oxo-2H-chromen-3-yl)-3-methylbenzamide: ^1^H & ^13^C NMR**


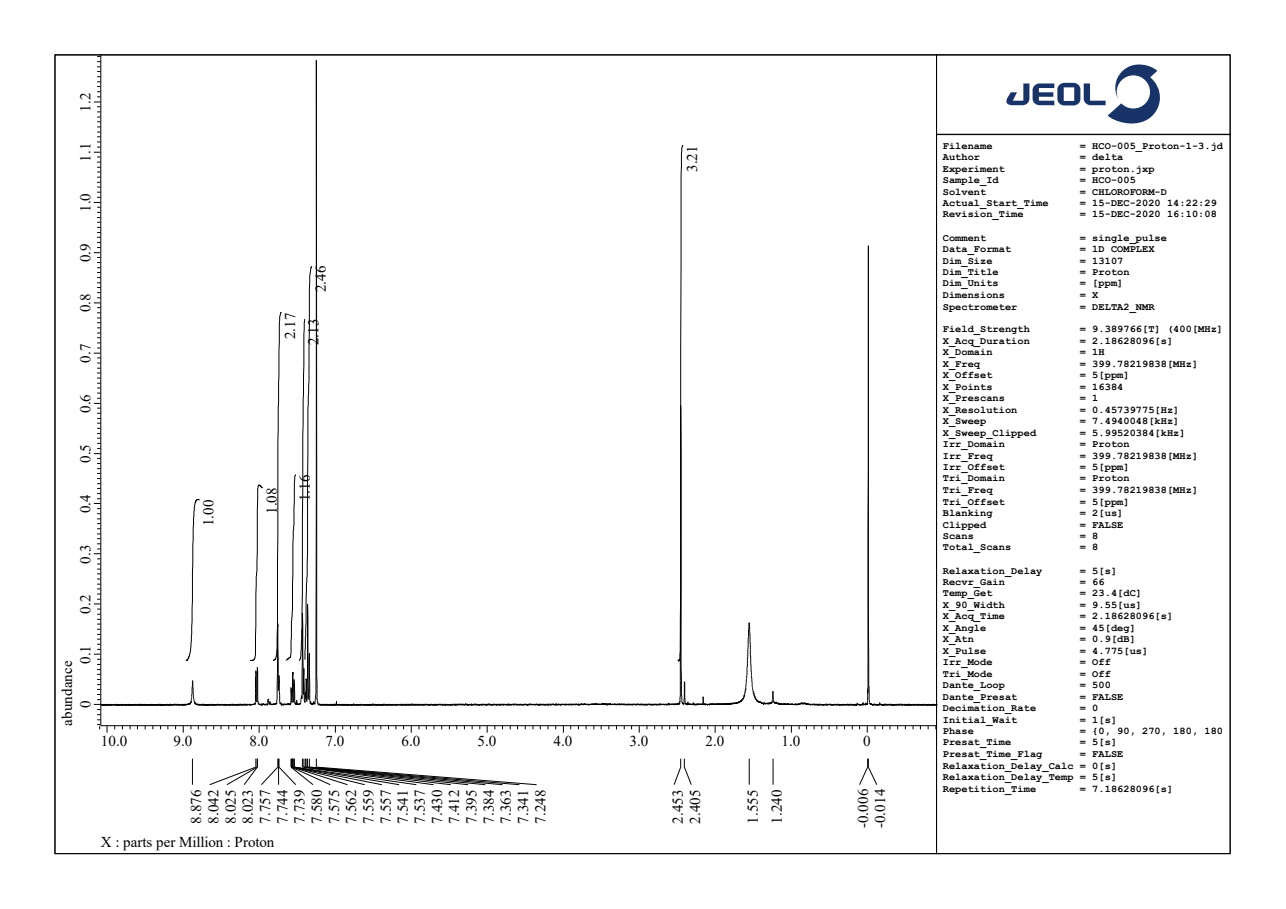

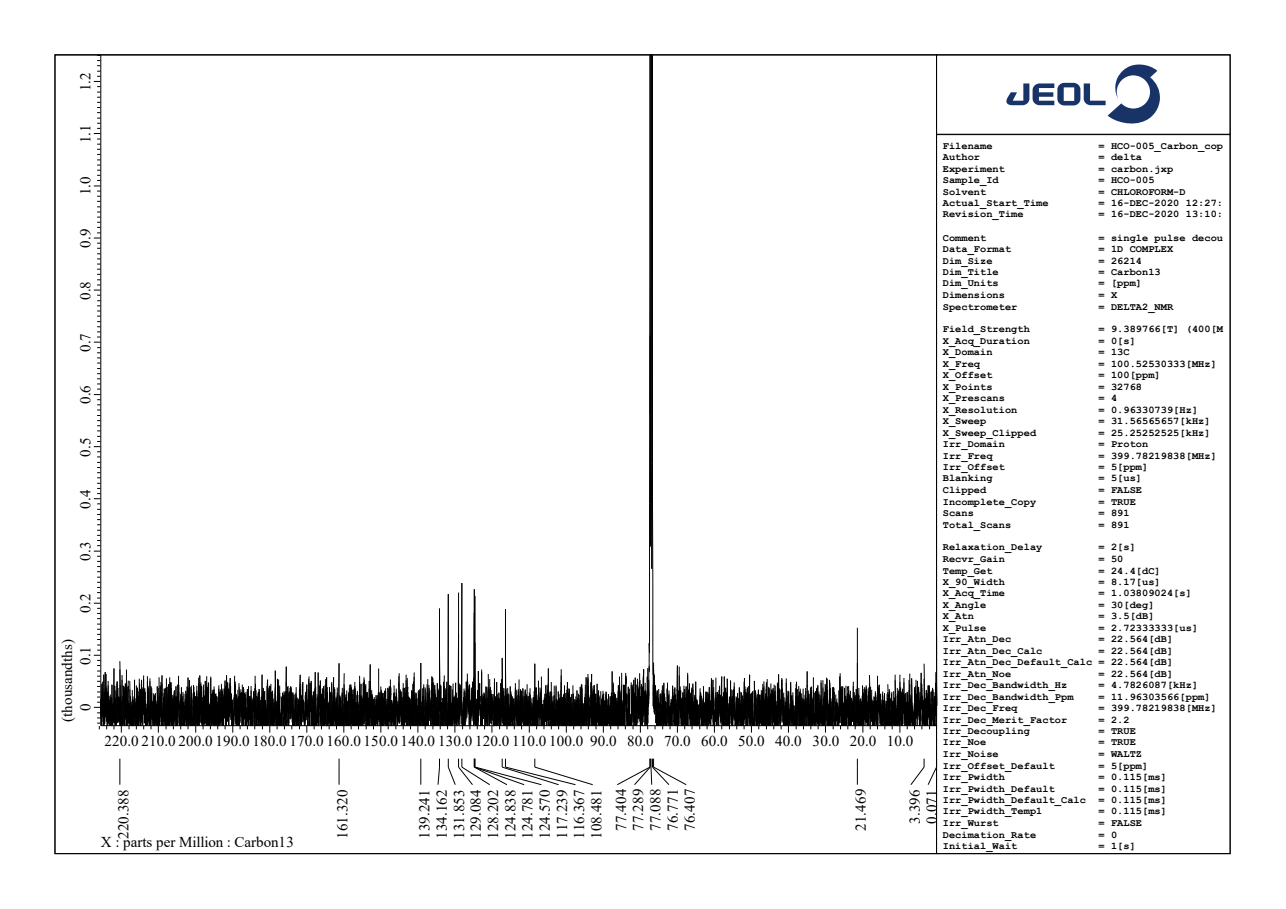


Supplementary Figure S5 (j)

**Compd. 4e: N-(4-hydroxy-2-oxo-2H-chromen-3-yl)-3-methylbenzamide: LC-MS Data**

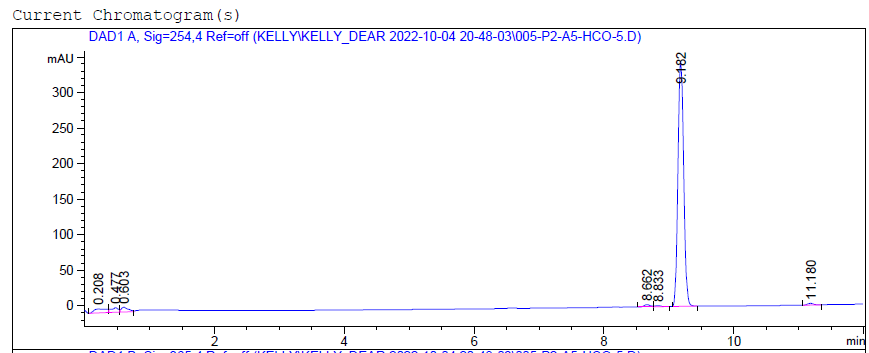


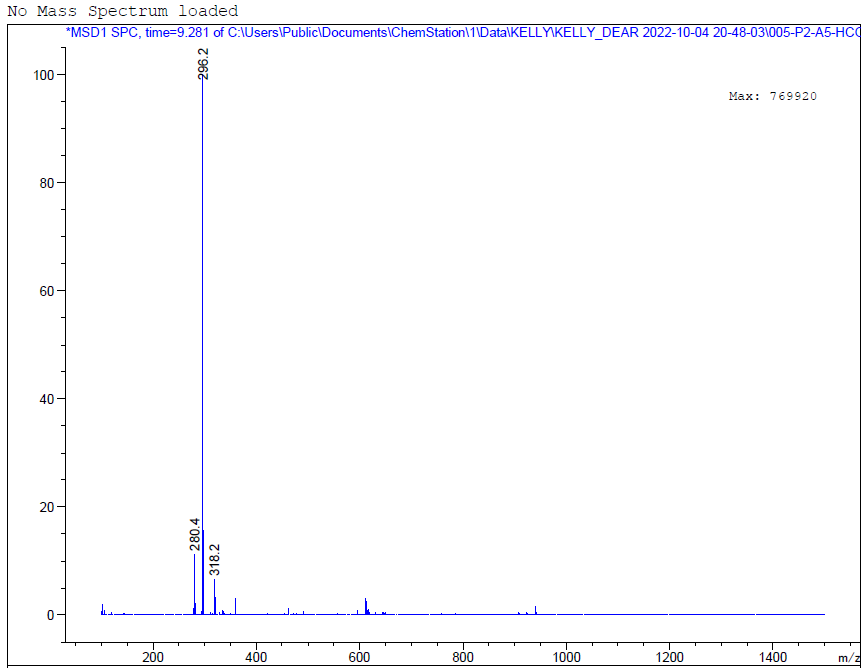


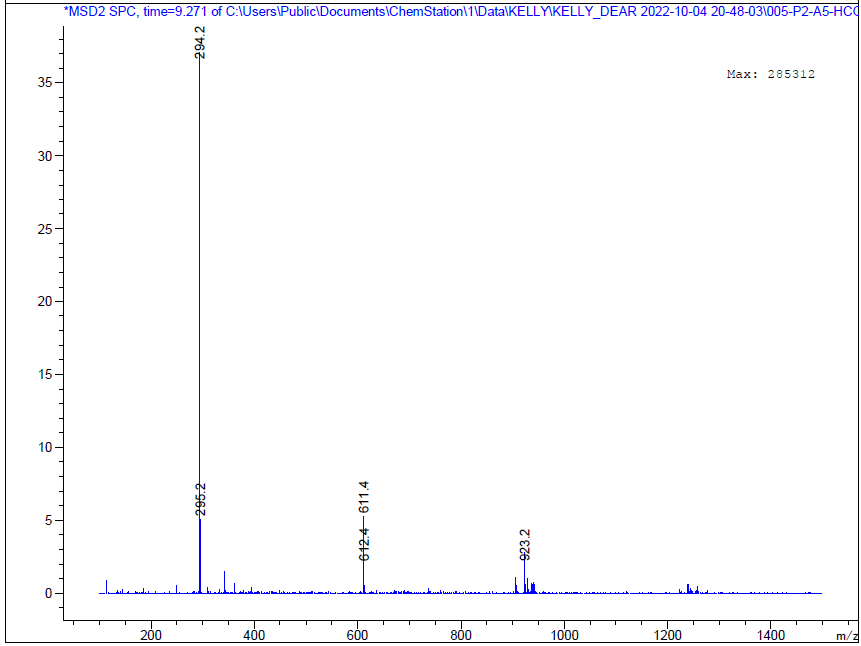


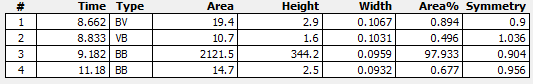


Supplementary Figure S5 (k)

**Compd. 4f: 4-chloro-N-(4-hydroxy-2-oxo-2H-chromen-3-yl)benzamide: ^1^H & ^13^C NMR**


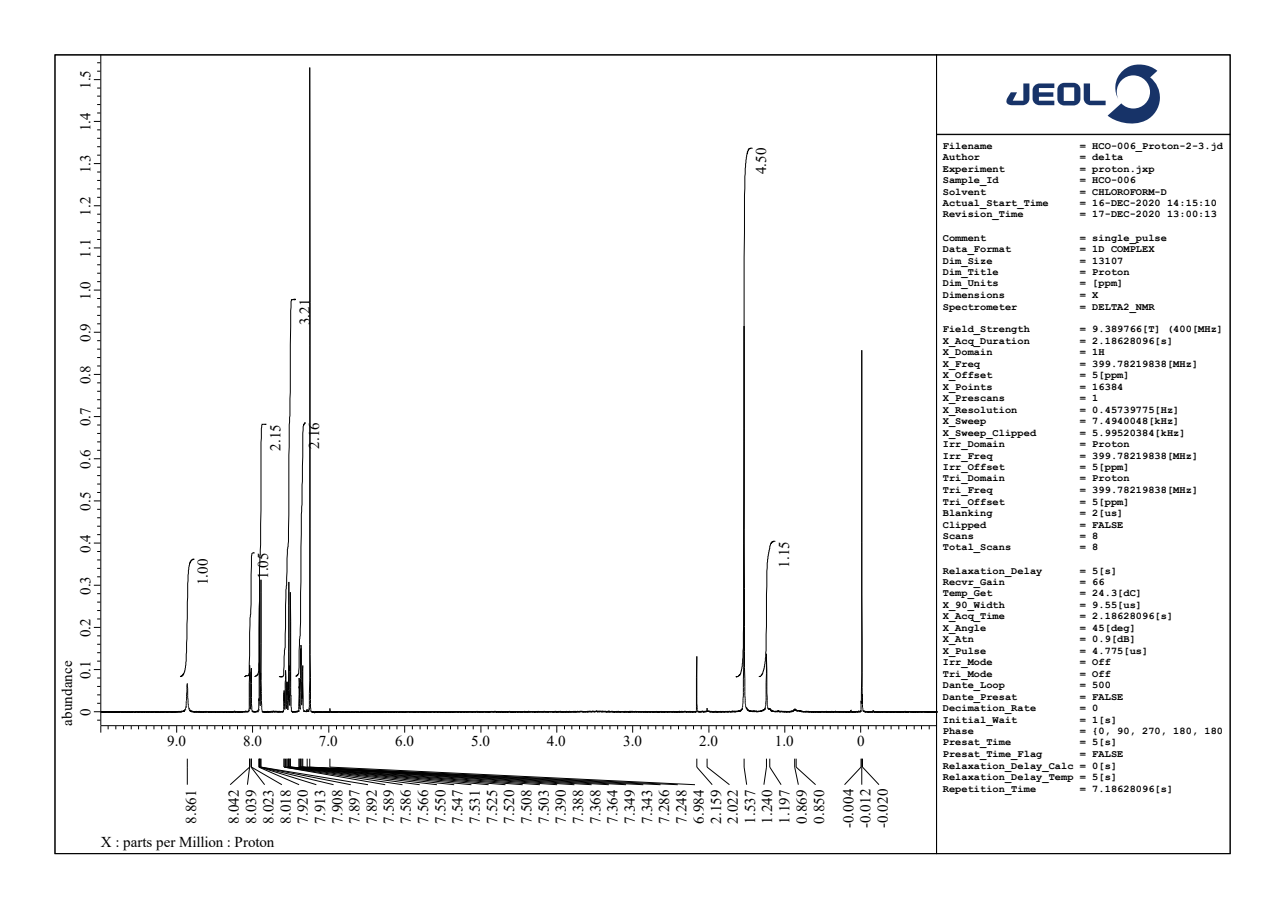

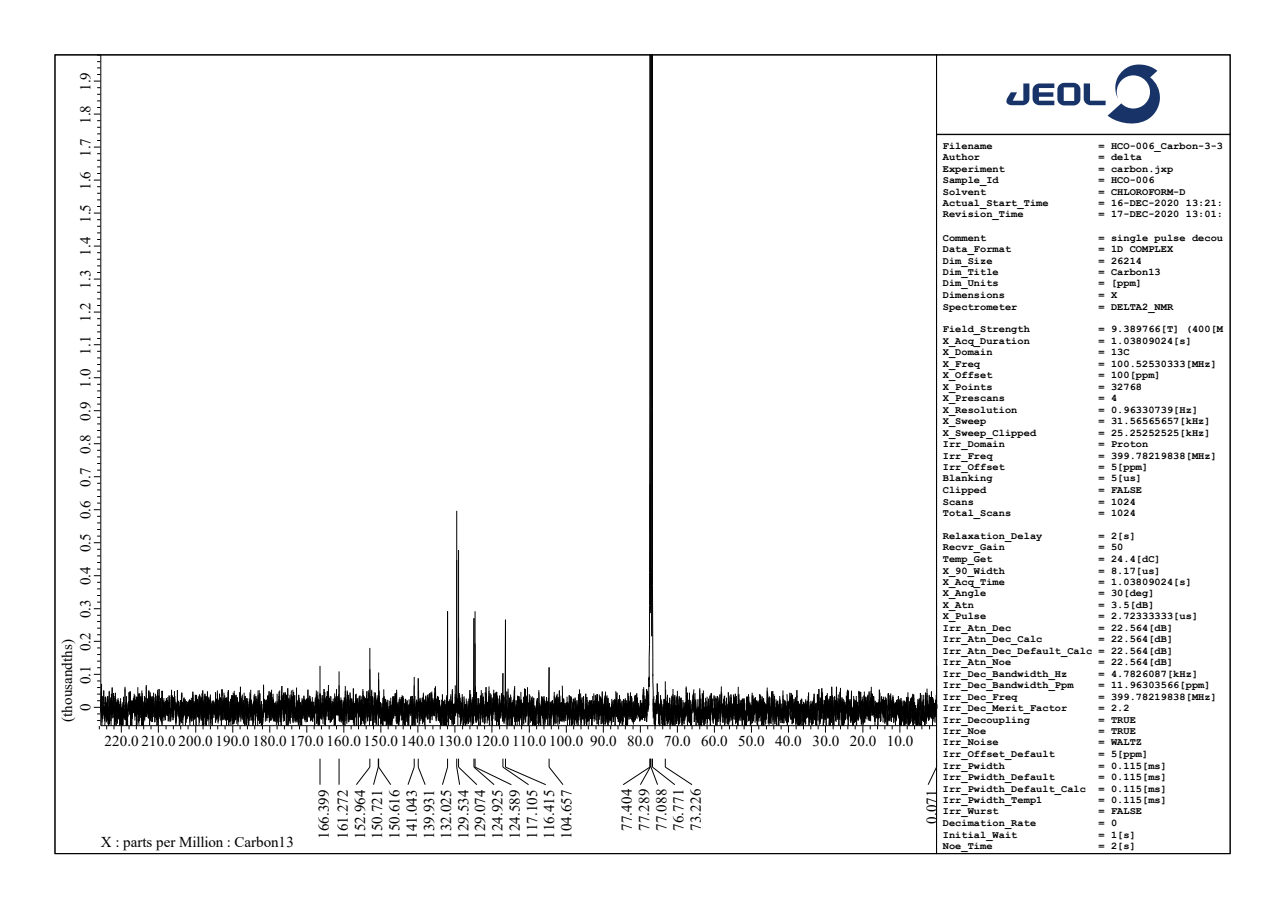


Supplementary Figure S5 (l)

**Compd. 4f: 4-chloro-N-(4-hydroxy-2-oxo-2H-chromen-3-yl)benzamide: LC-MS Data**

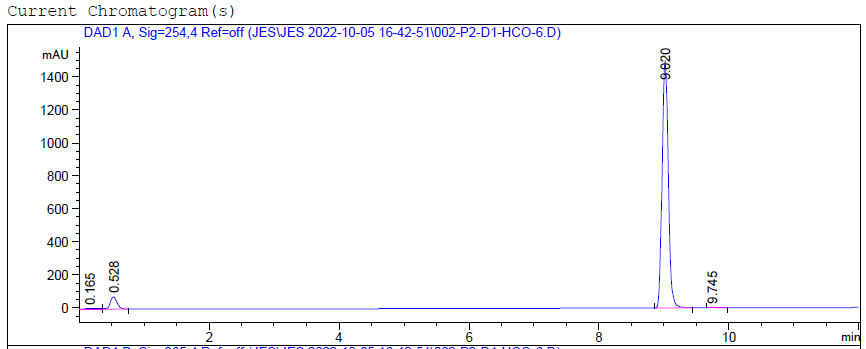


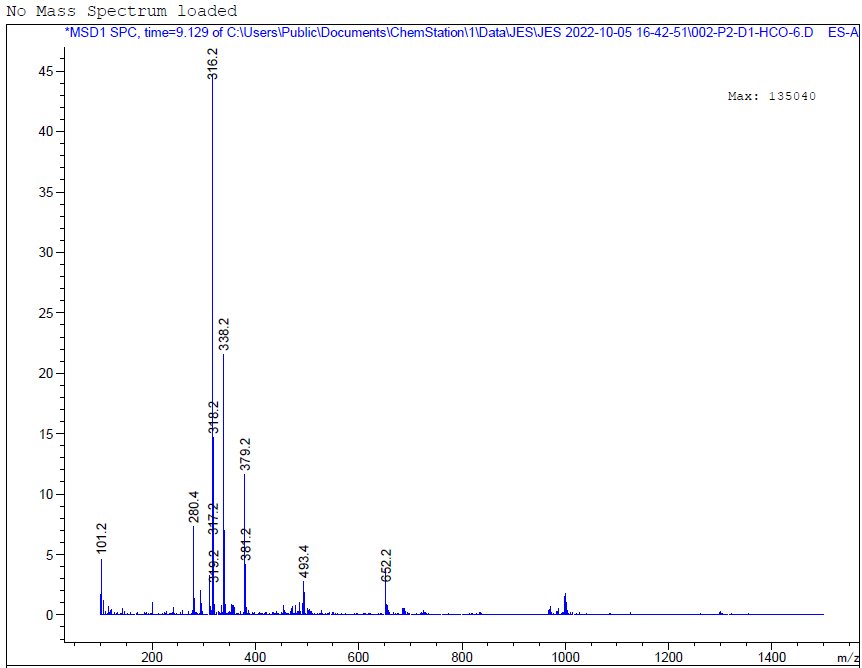


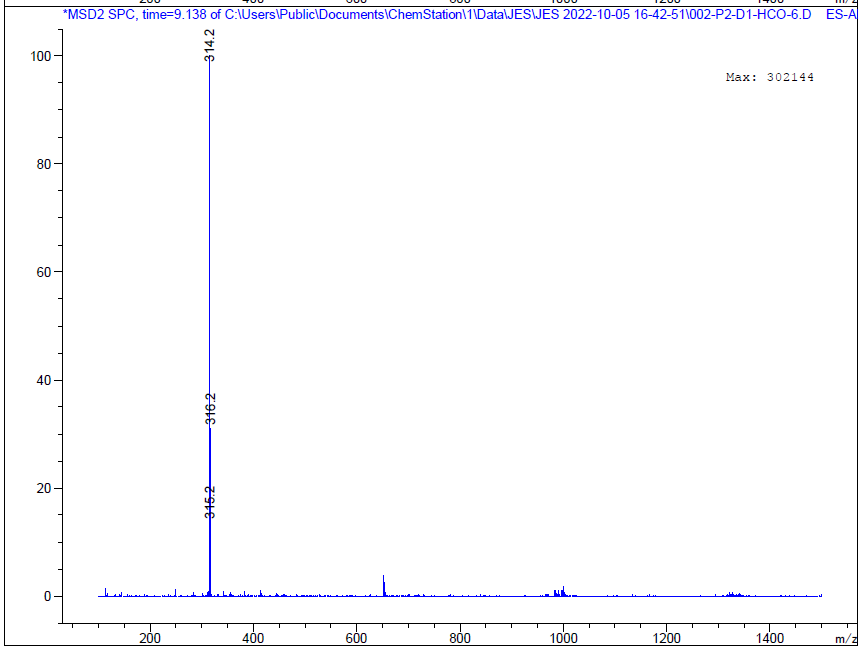


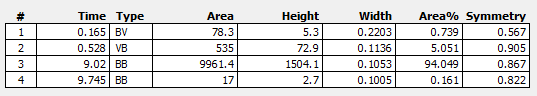


Supplementary Figure S5 (m)

**Compd. 4g:** **4-fluoro-N-(4-hydroxy-2-oxo-2H-chromen-3-yl)benzamide: ^1^H & ^13^C NMR**


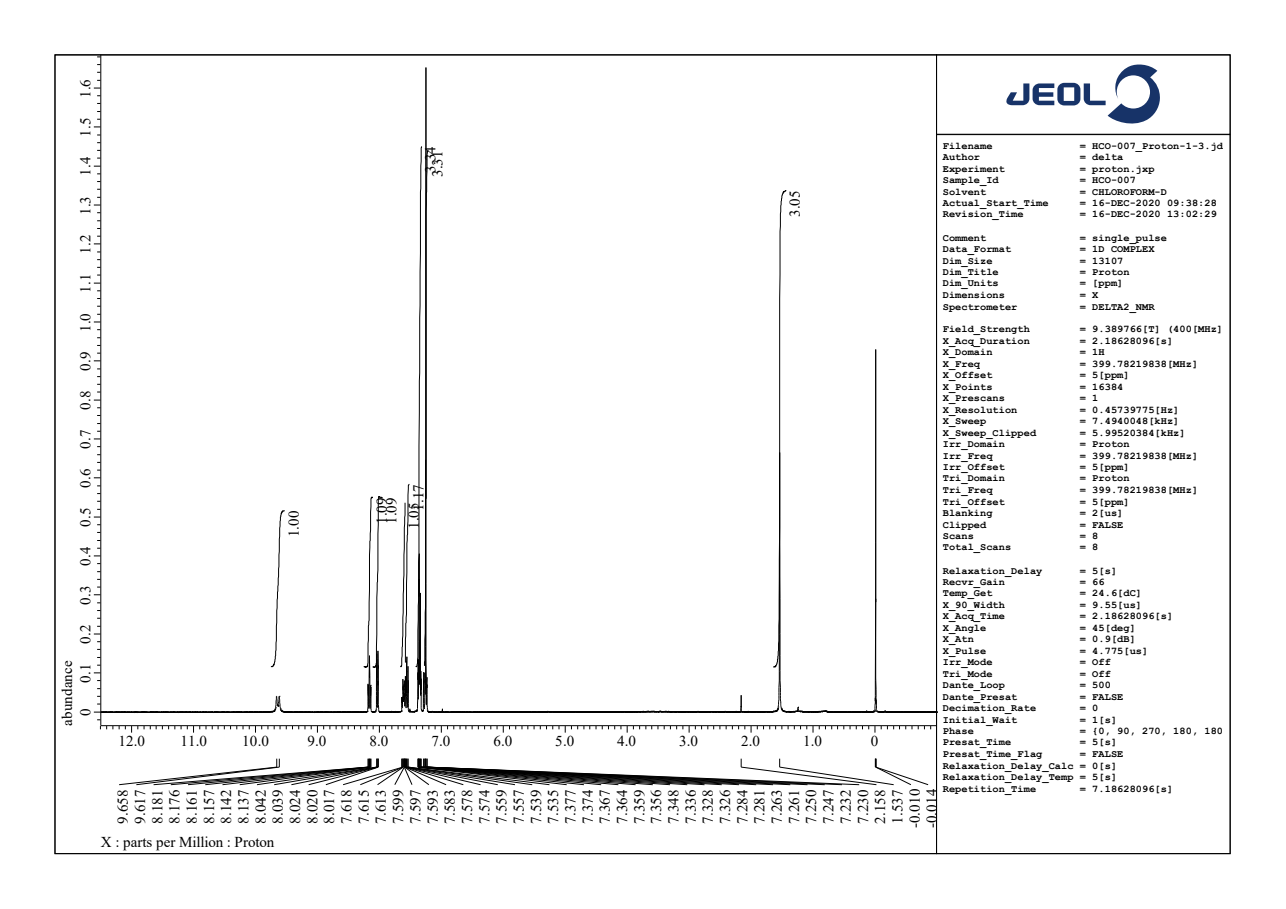

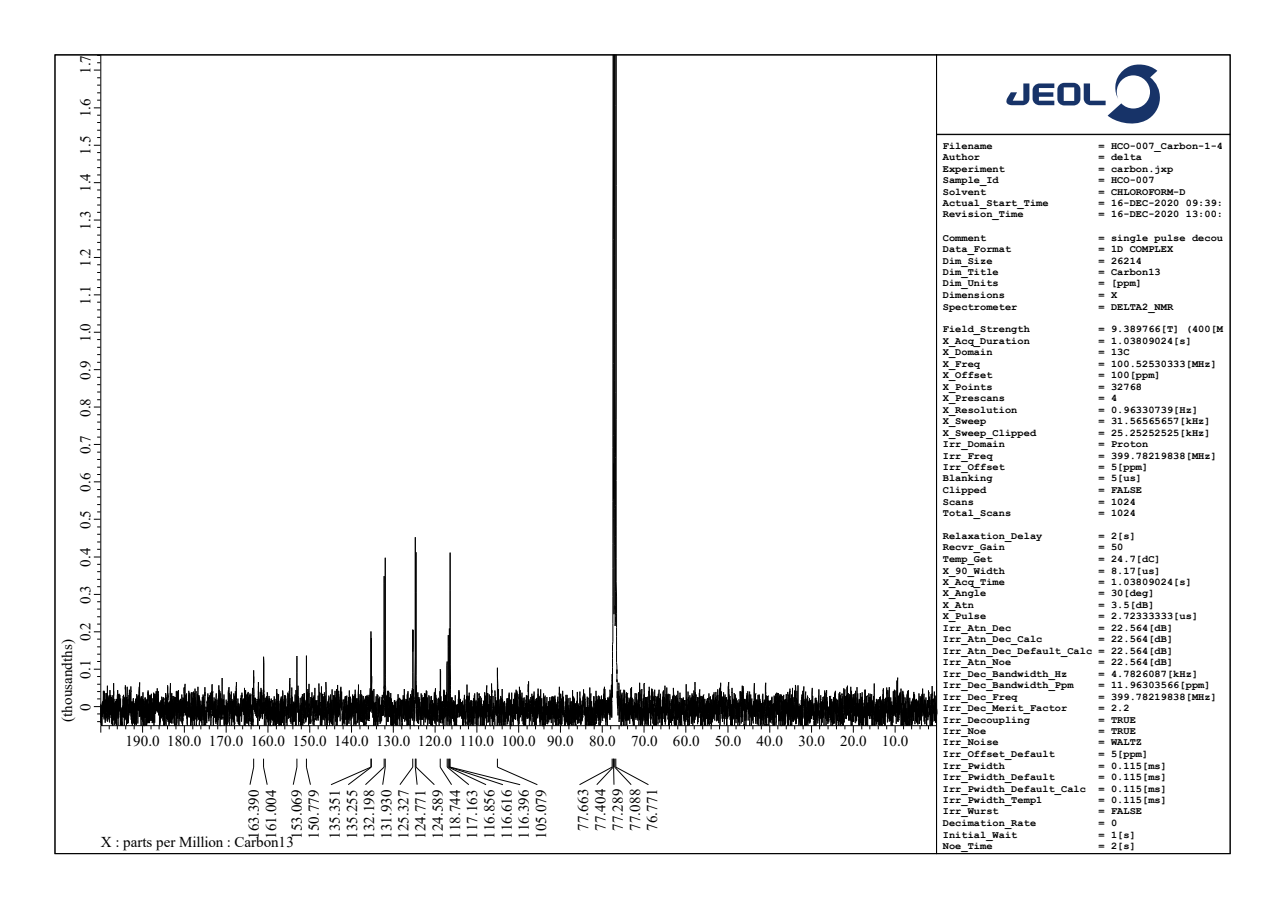


Supplementary Figure S5 (n)

**Compd. 4g: 4-fluoro-N-(4-hydroxy-2-oxo-2H-chromen-3-yl)benzamide: LC-MS Data**

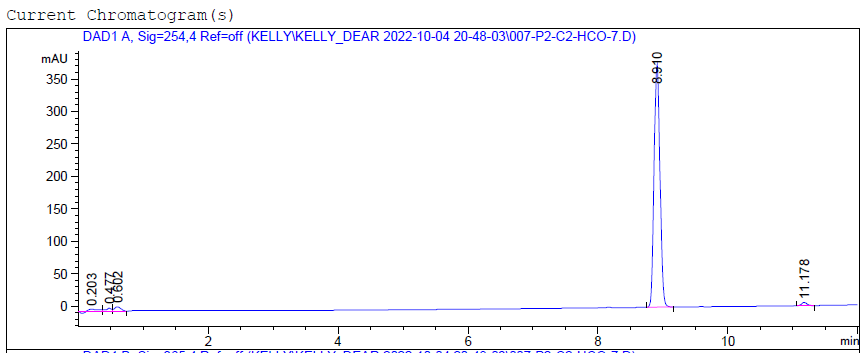


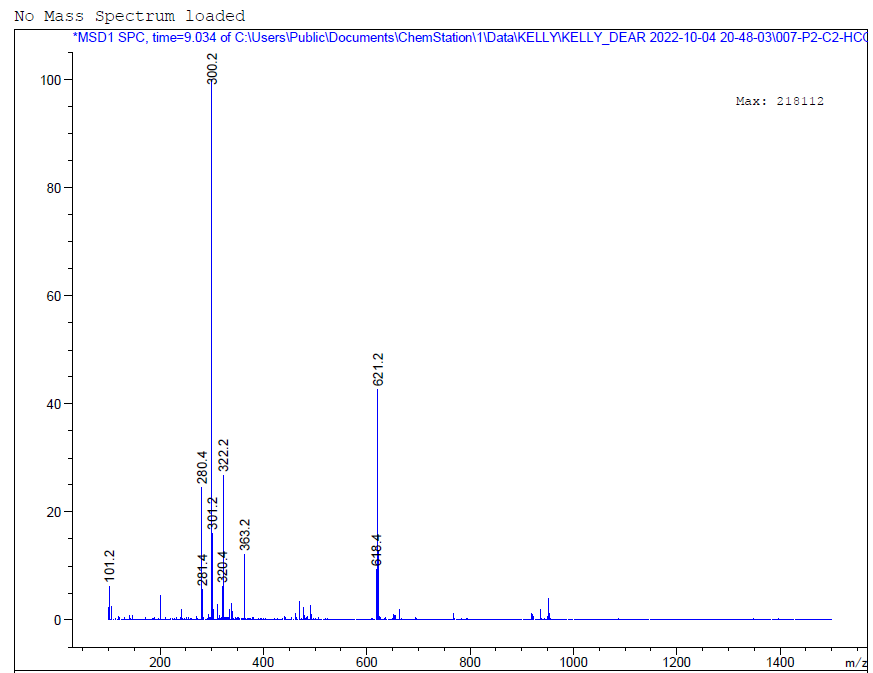


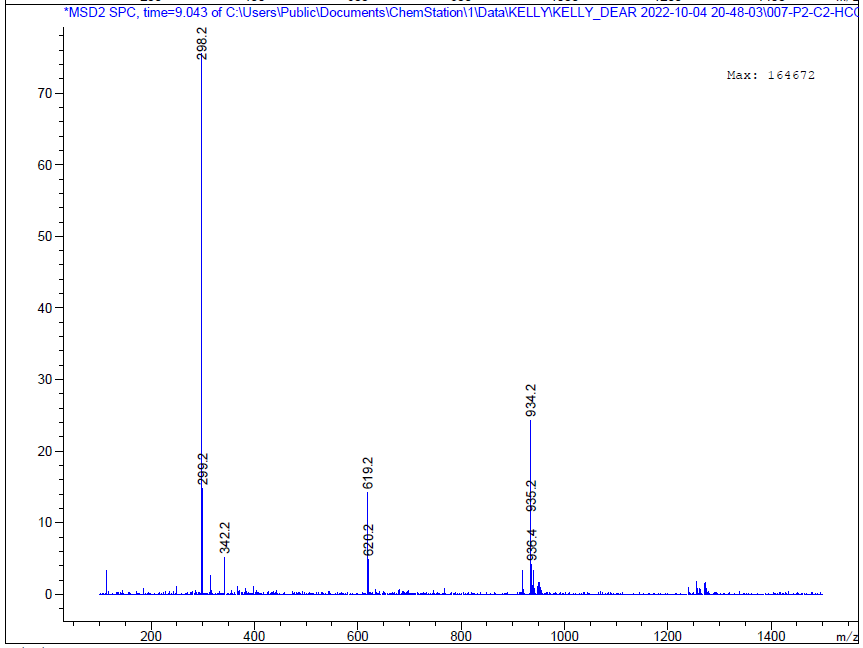


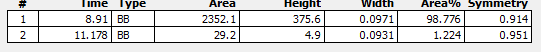


Supplementary Figure S5 (o)

**Compd. 4h: 3-chloro-N-(4-hydroxy-2-oxo-2H-chromen-3-yl)benzamide: ^1^H & ^13^C NMR**


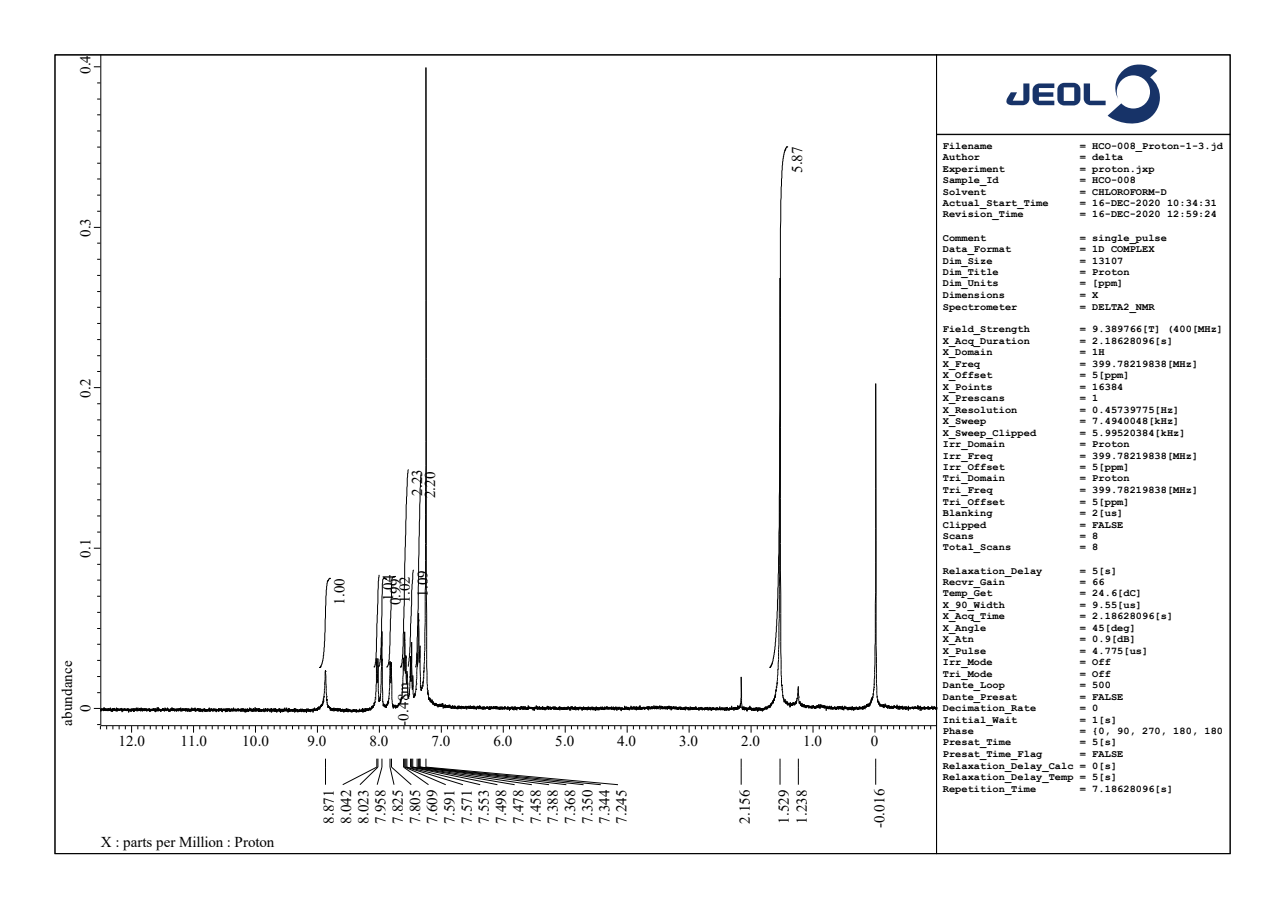

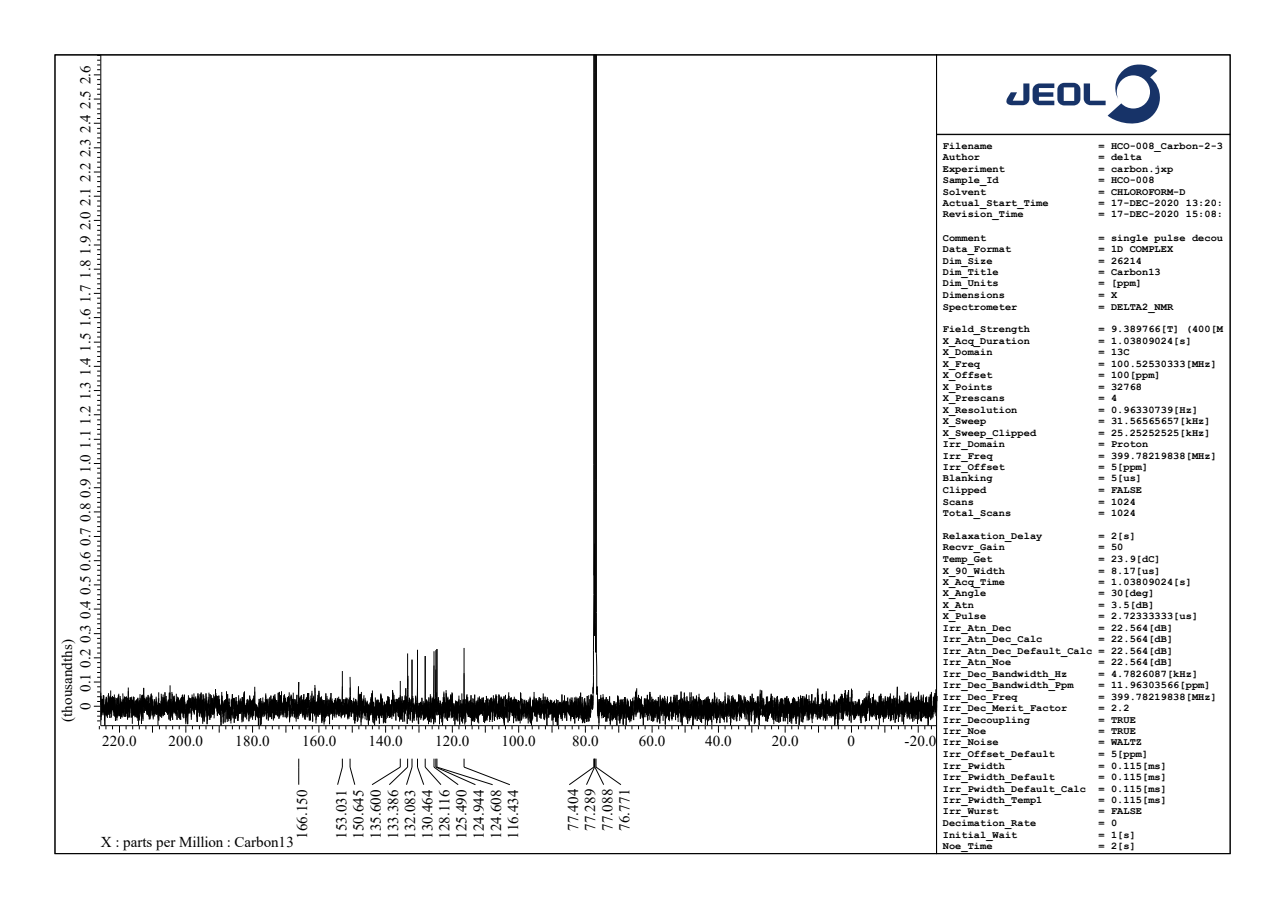


Supplementary Figure S5 (p)

**Compd. 4h: 3-chloro-N-(4-hydroxy-2-oxo-2H-chromen-3-yl)benzamide: LC-MS Data**

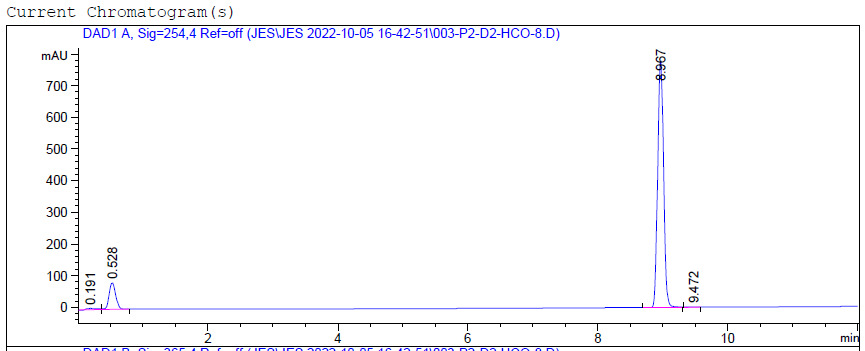


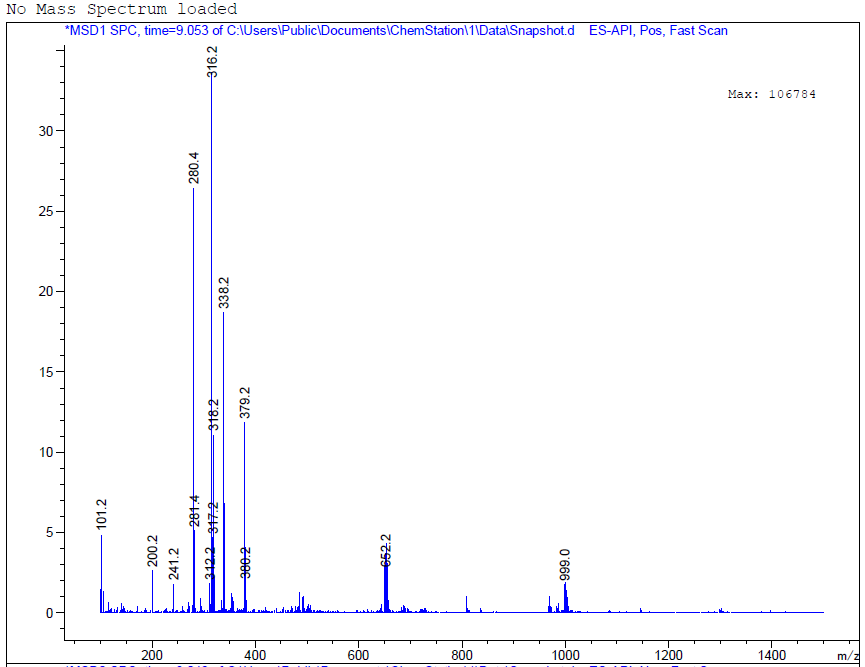


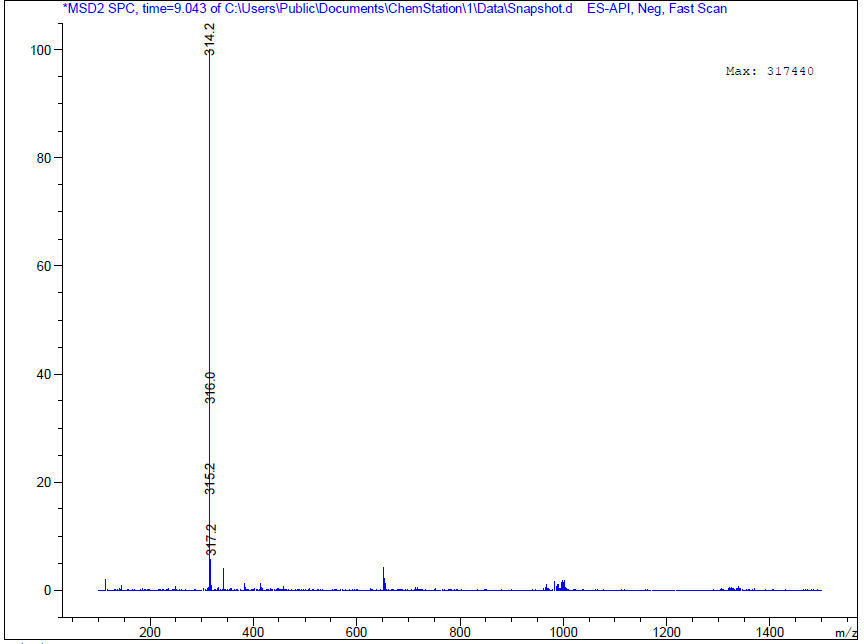


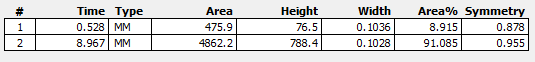


Supplementary Figure S5 (q)

**Compd. 4i: N-(4-hydroxy-2-oxo-2H-chromen-3-yl)-[1,1'-biphenyl]-4-carboxamide:^1^H & ^13^C NMR**


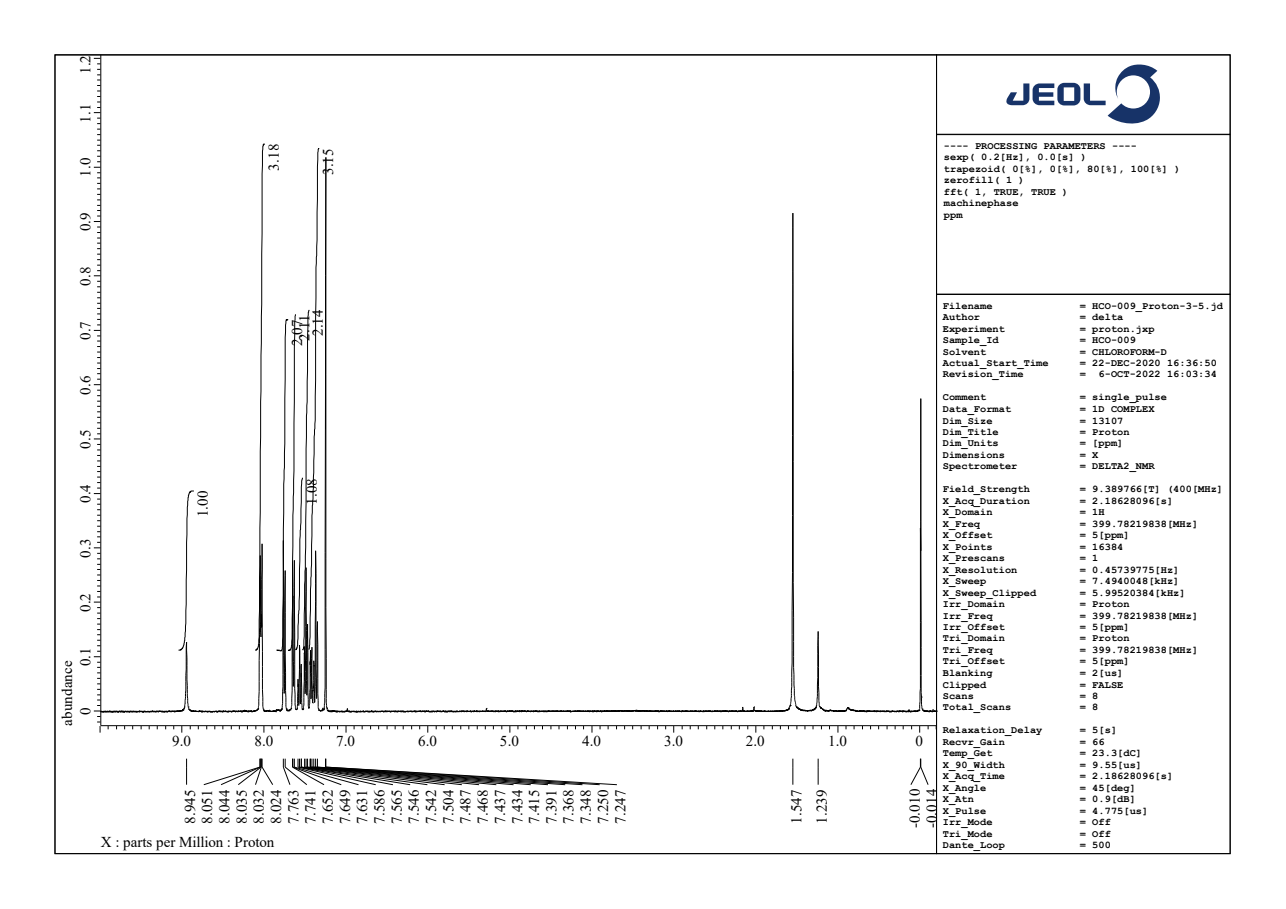


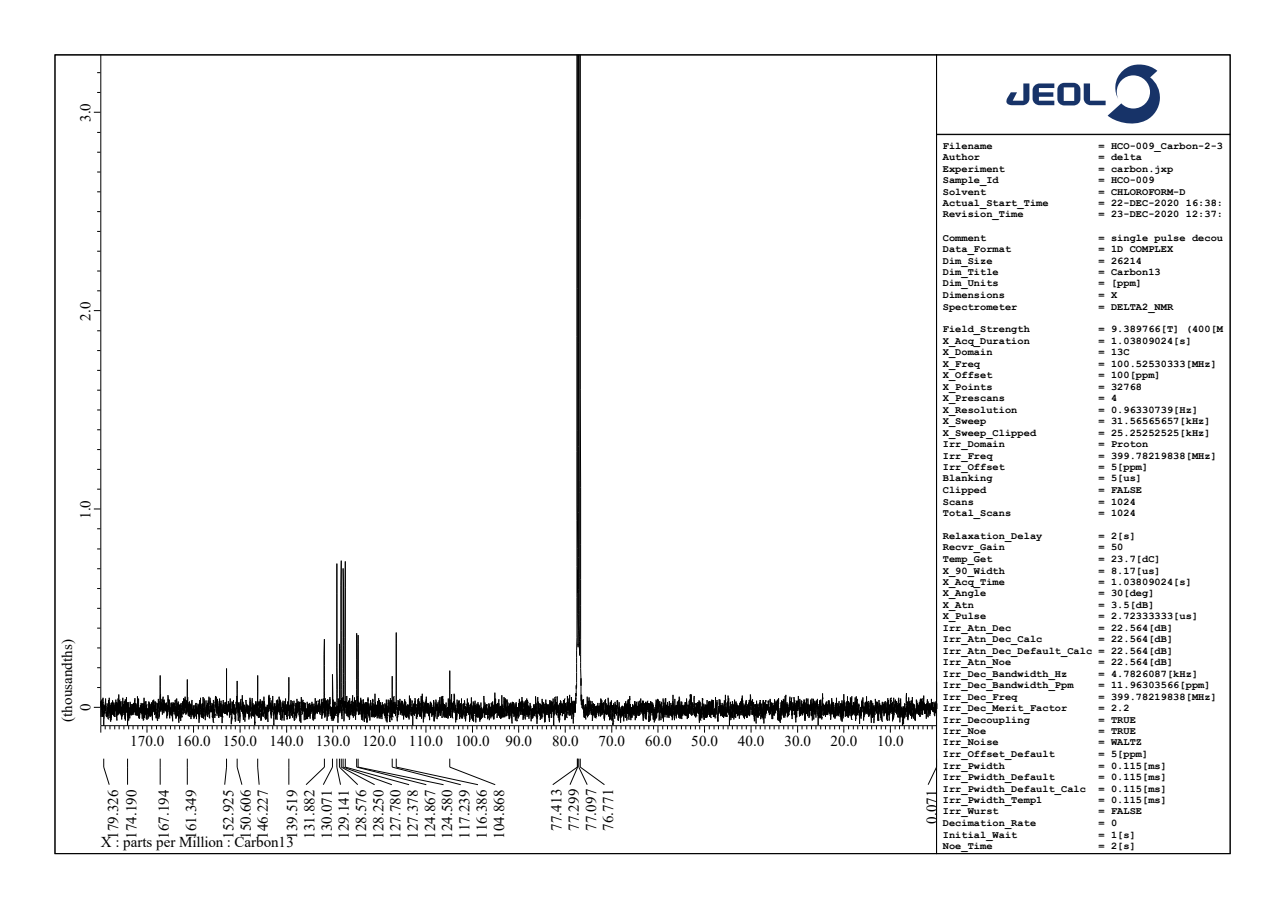


Supplementary Figure S5 (r)

**Compd. 4i: N-(4-hydroxy-2-oxo-2H-chromen-3-yl)-[1,1'-biphenyl]-4-carboxamide: LC-MS Data**

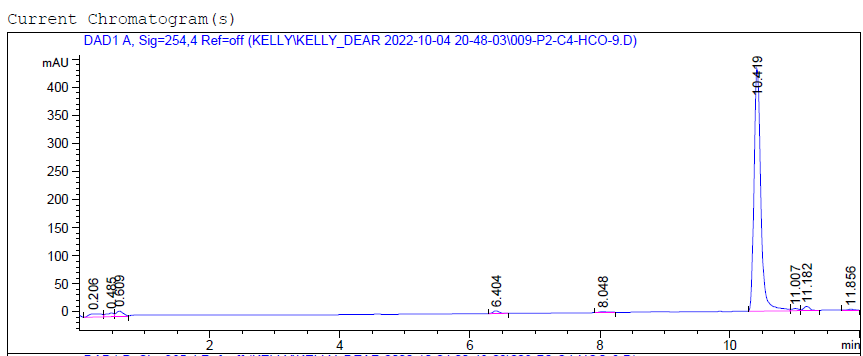


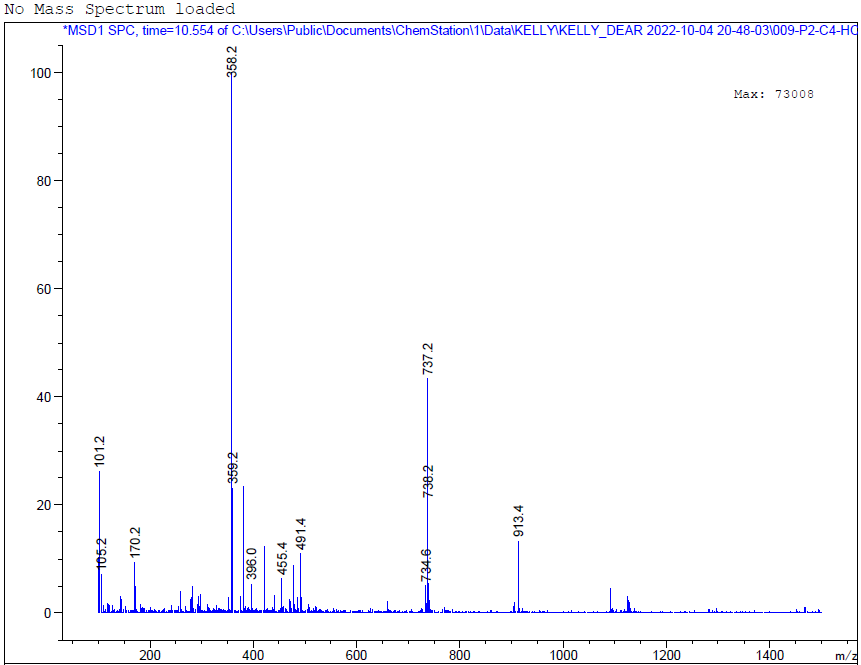


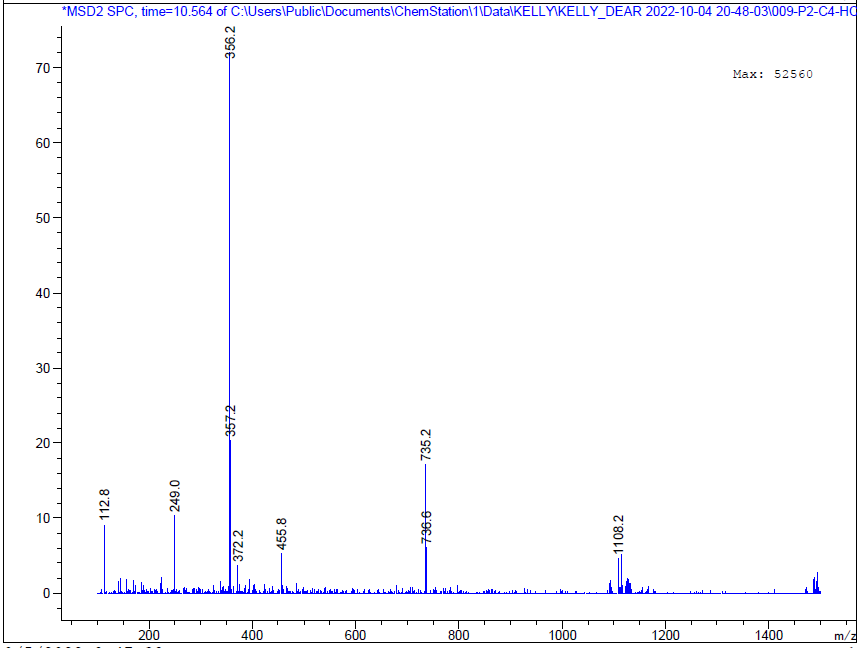


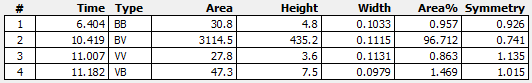


Supplementary Figure S5 (s)

**Compd. 4j: 2-fluoro-N-(4-hydroxy-2-oxo-2H-chromen-3-yl)benzamide: ^1^H & ^13^C NMR**


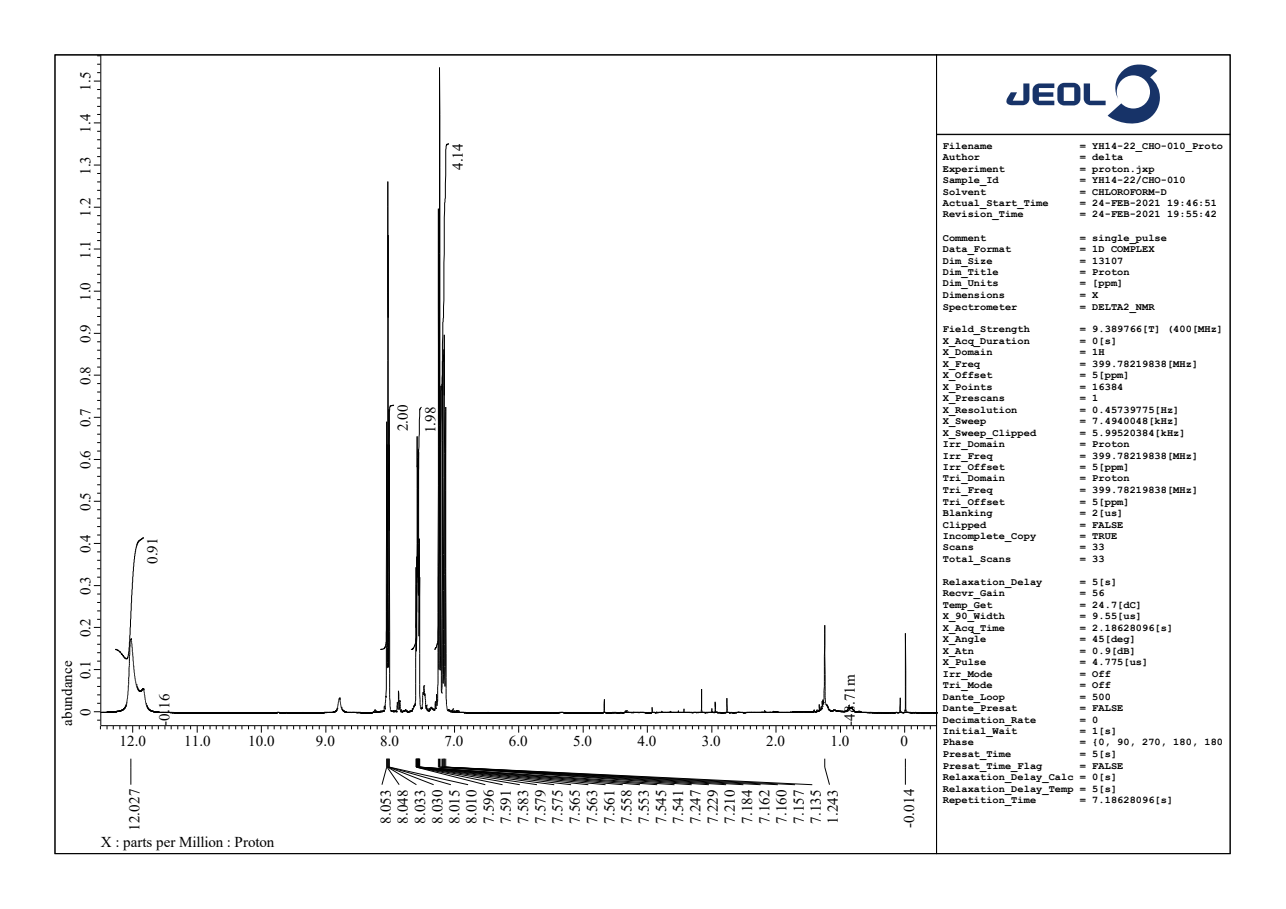


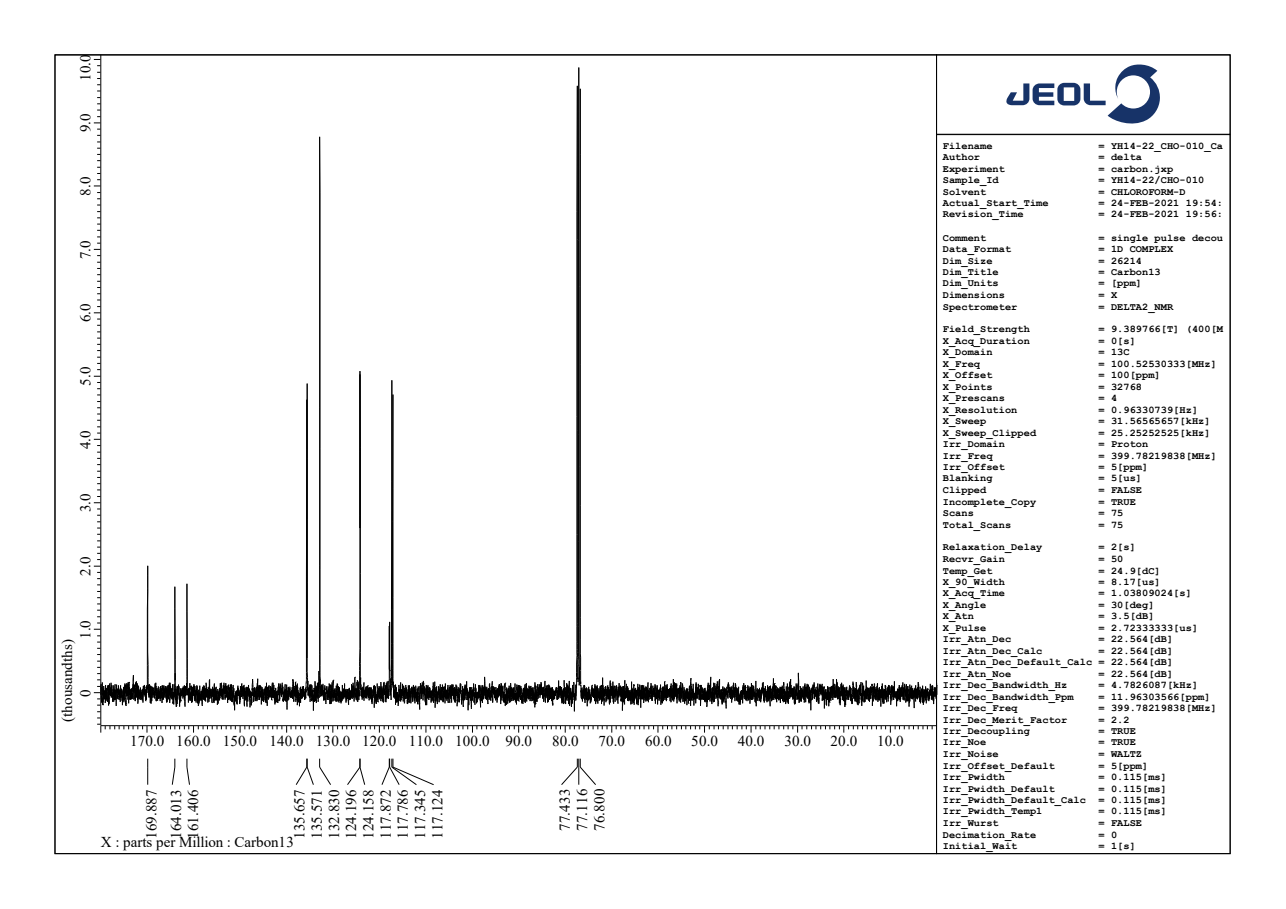


Supplementary Figure S5 (t)

**Compd. 4j: 2-fluoro-N-(4-hydroxy-2-oxo-2H-chromen-3-yl)benzamide: LCMS Data**

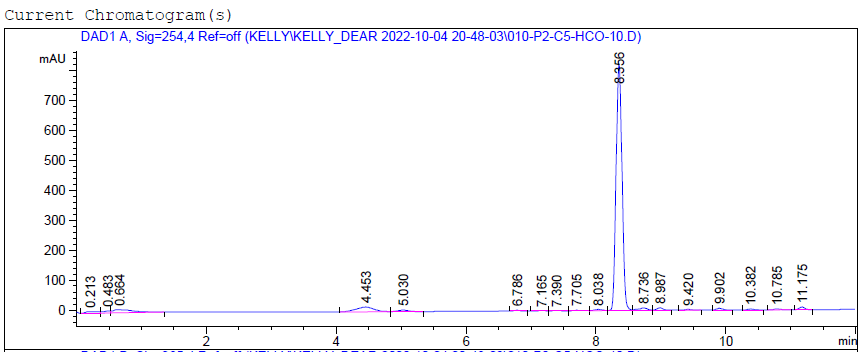


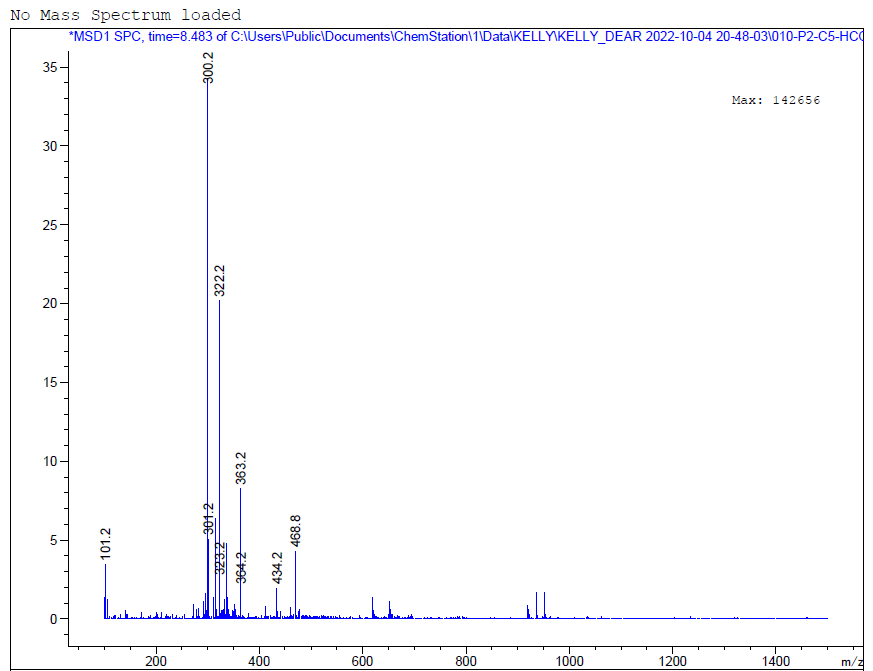


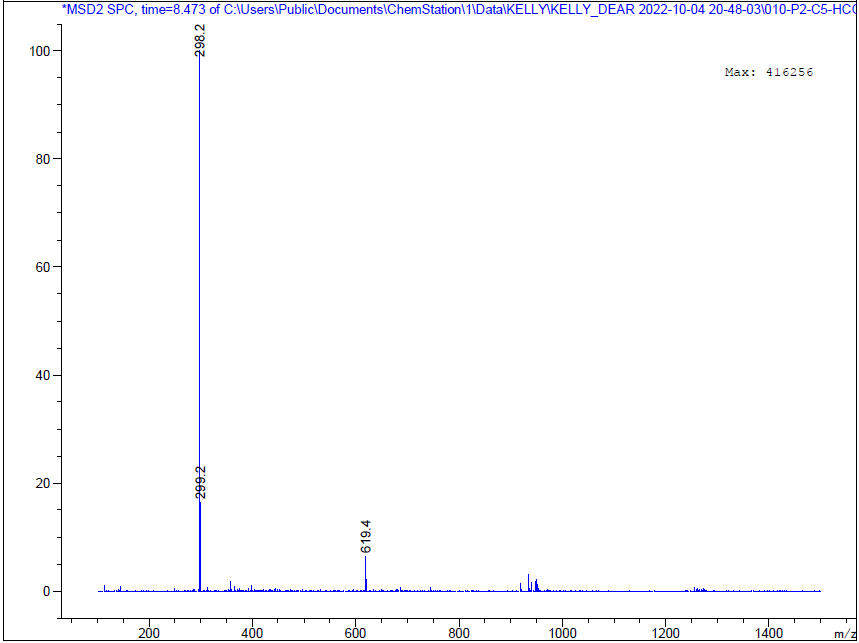


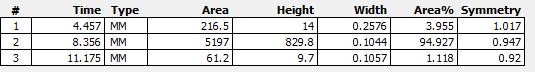

Supplement: Supplementary file 1 — Supplementary Figures. [file 41598_2022_26212_MOESM1_ESM.docx]
